# Growth environment and organ specific variation in in-vitro cytoprotective activities of *Picea mariana* in PC12 cells exposed to glucose toxicity: a plant used for treatment of diabetes symptoms by the Cree of Eeyou Istchee (Quebec, Canada)

**DOI:** 10.1186/s12906-019-2550-4

**Published:** 2019-06-18

**Authors:** Ashleigh D. Downing, Hoda M. Eid, Andrew Tang, Fida Ahmed, Cory S. Harris, Pierre S. Haddad, Timothy Johns, John T. Arnason, Steffany A. L. Bennett, Alain Cuerrier

**Affiliations:** 1 Jardin botanique de Montreal, Institut de recherche en biologie végétale, Université de Montréal, 4101, rue Sherbrooke Est, Montreal, (Québec) H1X 2B2 Canada; 2 Department of Pharmacology, Natural Health Products and Metabolic Diseases Laboratory, Université de Montréal, Montreal, QC Canada; 3 Department of Pharmacognosy, Faculty of Pharmacy, Beni-Suef University, Beni-Suef, Egypt; 4 Department of Biology, Laboratory for the Analysis of Natural and Synthetic Environmental Toxins, University of Ottawa, Ottawa, ON Canada; 5 School of Dietetics and Human Nutrition, McGill University, Montreal, Canada; 6 Department of Biochemistry, Microbiology, and Immunology,, University of Ottawa, Ottawa, Canada

**Keywords:** Traditional medicine, PC12-AC, UPLC-QTOF, DPPH, Phenolics, Stilbenes, Glucose toxicity

## Abstract

**Background:**

The Cree of Eeyou Istchee (James Bay area of northern Quebec) suffer from a high rate of diabetes and its complications partly due to the introduction of the western lifestyle within their culture.

As part of a search for alternative medicine based on traditional practice, this project evaluates the biological activity of *Picea mariana* (Mill.) Britton, Sterns & Poggenb. needle, bark, and cone, in preventing glucose toxicity to PC12-AC cells in vitro (a diabetic neurophathy model) and whether habitat and growth environment influence this activity.

**Methods:**

Three different organs (needle, bark, and cone) of *P. mariana* were collected at different geographical locations and ecological conditions and their 80% ethanolic extracts were prepared. Extracts were then tested for their ability to protect PC12-AC cells from hyperglycaemic challenge at physiologically relevant concentrations of 0.25, 0.5, 1.0 and 2.0 μg/mL. Folin-Ciocalteu method was used to determine the total phenolic content of *P. mariana* extracts.

**Results:**

All extracts were well-tolerated in vitro exhibiting LD_50_ of 25 *μ*g/mL or higher. Extracts from all tested organs showed a cytoprotective concentration-dependent response. Furthermore, the cytoprotective activity was habitat- and growth environment-dependent with plants grown in bog or forest habitats in coastal or inland environments exhibiting different cytoprotective efficacies. These differences in activity correlated with total phenolic content but not with antioxidant activity. In addition, this paper provides the first complete Ultra-Performance Liquid Chromatography-quadrupole time-of-flight (UPLC-QTOF) mass spectrometry analysis of *Picea mariana*’s bark, needles and cones.

**Conclusions:**

Together, these results provide further understanding of the cytoprotective activity of Canadian boreal forest plants identified by the Cree healers of Eeyou Istchee in a cell model of diabetic neuropathy. Their activity is relevant to diabetic peripheral neuropathic complications and shows that their properties can be optimized by harvesting in optimal growth environments.

**Electronic supplementary material:**

The online version of this article (10.1186/s12906-019-2550-4) contains supplementary material, which is available to authorized users.

## Background

In recent years, there has been increasing recognition of the deteriorating health status of Canadian First Nation peoples with respect to chronic diseases [1, 2]. Among the chronic diseases afflicting these populations, diabetes has stood out as a literal epidemic [3–5]. Low levels of compliance due to treatment incompatibility have led to high levels of severe complications in these populations [3, 6–8].

*Picea mariana* (Mill.) Britton, Sterns & Poggenb., commonly known as black spruce or Inaatuk in Cree has been used as a folk remedy for skin and soft tissues conditions and as analgesic [9–11]. Poultice of inner bark has been applied for infected inflammations [10]. Infusion of bark has been used for pain relief [9] and the gum soaked in hot water has been used in burn dressing or smeared on painful areas of the body [11]. Decoction of needles has been applied to cure skin sores and to speed wound healing [12].

The proposed solution to the rejection of western medication, as part of the CIHR Team in Aboriginal Antidiabetic Medicines (TAAM), is to make traditional treatment options readily available alongside conventional treatment [7, 13]. To accomplish this goal, semi-structured ethnobotanical surveys have been conducted in Misstisini, Whapmagoostui [14, 15], Waskaganish and Nemaska [16]. Using the Syndromic Importance Value (SIV) calculation, *P mariana* emerged in ethnobotanical surveys as a top ranking plant [15]. Specifically, it is mentioned as a possible treatment for symptoms related to diabetic neuropathy (pain, loss of sensation which increases risk of wounds to the extremities, slow-healing wounds and greater risk of wound infections due to weakened immune response) [14, 15].

To date, only antidiabetic activities of a cone extract have been investigated. The extract showed high antioxidant activity, insulin sensitizing and glitazone-like effects. It also conferred protection to PC12 cells, a model of peripheral neuronal precursors from high glucose insult [14, 17, 18]. Given these data and that extracts from needles of *Picea glauca* (Moench) Voss (a closely related species) also protect PC12 cells from hyperglycemic challenge [19], we compared the cytoprotective efficacy of extracts prepared from three different organs (needle, bark and cone) of *P. mariana* collected at different geographical locations and ecological conditions. Our primary goal was to establish whether cytoprotective activity was dependent upon habitat and growth environment and to elucidate what the ideal collection conditions might be for this plant. Here we describe how cytoprotective and mitogenic activities differ between extracts sampled from coastal and inland populations (Waskaganish to Mistissini) in areas where bog (low land) meets forest (high land). Six of nine Eeyou Istchee communities across a varied northern Quebec landscape have already participated in ethnobotanical studies that may lead to the incorporation of traditional pharmacopeia into clinics. Communities are situated inland (Mistissini, Nemaska, Oujé-Bougoumou) or on the coast of James or Hudson Bay (Waskaganish, Wemindji, and Whapmagoostui). The evaluation of local harvesting is essential to determine whether plant organs will yield similar benefits under different growing conditions, knowing especially that this region is vast. The development of this research question has come directly from the elders of Eeyou Istchee who emphasized the importance of plant selection and regional variation.

## Methods

### Plant material

**Fig. 1:**
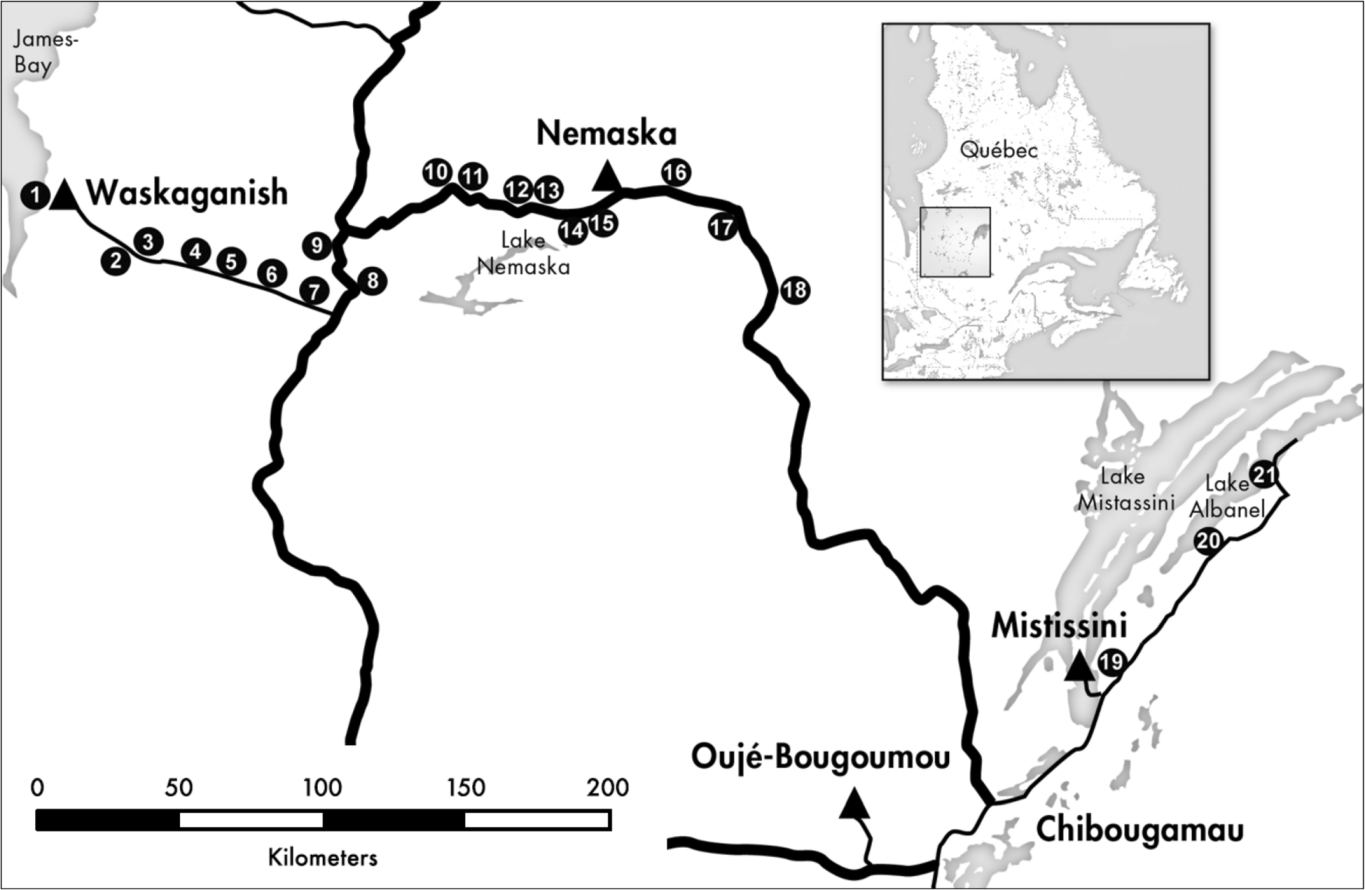
Map representing plant sampling locations (white number in black circle). The markers represent both the high and low land locations since they were next to each other. For example the circle labelled “3” represents bog and forest populations. At this location materials for B3N, B3B, B3C, F3N, F3B and F3C were collected. Cree communities are represented by a ▲ symbol. This map was simplified using photoshop, from “The Far North Nunavik and James Bay” tourist map which was sourced from the Base de données géographiques et administratives du Québec (BDGA)

*P. mariana* organs (needle, bark and cone) were harvested in proximity to the Cree communities of Waskaganish, Nemaska and Mistissini located within the 50° and 51° N latitudes (Quebec, Canada). Twenty-one populations were selected (Fig. 1). Plants were harvested at the boundaries of high land (forest) and low land (bog) to enable collection from two distinct habitats (bog or forest) within the same geographical growth environment (measured as proximity to the James Bay coast). Growth environment was considered coastal if it was < 50 km from the coast and inland if further. To ensure that habitats were distinct, bog stands were defined as trees growing in > 30 cm sphagnum moss. Forests were defined as having trees growing in organic soil with < 20 cm sphagnum moss ground cover. Separate populations had to be at least 5 km apart and in most cases were much further in order to sample across all three communities. Needles and bark were harvested from 10 trees within a 50 m radius for each habitat. Cones were collected from the same trees when available however, they were often unreachable or not present. All bulk materials were air dried upon collection and dried to completion in a plant drier at 40 °C prior to shipment. All samples were collected and dried within a few weeks. The dried samples were extracted at the University of Ottawa. Voucher specimens were deposited at the Marie-Victorin Herbarium (MT) located at the Jardin botanique de Montréal (PM001 to PM040).

### Extract preparation

Twenty populations of needles were extracted. Pooled samples were made by weighing out 2 g of material from each tree (10x) resulting in a total of 2 g per location. The 2 g sample was placed in a magic bullet blender (Homeland Housewares) along with 20 mL of 80% ethanol (10 mL/g material) and collected in 50 mL Fisher falcon tubes. After blending, the sample was sonicated for 20 min and the first extraction mixture was stored at 4 °C for 1 week. The ethanol extract was centrifuged for 15 min at 1811 g and the liquid supernatant was removed by pipette and stored at 4 °C. Ten mL of fresh 80% ethanol was added to the pellet, sonicated and stored at 4 °C. The second supernatant was then removed and added in the same fashion as the first. Alcohol was then removed from the pooled supernatant using a Labconco Centrivap (Fisher Scientific, Ottawa, Canada) at 40 °C for 72 h. The alcohol-free extract was lyophilized using a freeze dryer (Edwards Pirani 501, Fisher Scientific, Ottawa, Canada) for 72 h. The result was a dry stable extract that was homogenized and stored in a freezer at − 20 °C. Nine of the 20 populations were included for the analysis of bark extracts. It was necessary in the case of bark and cone to grind samples in a Wiley mill (2 mm mesh) tree by tree before evenly pooling the populations. As before 2 g of the pooled sample was processed using the same protocol as for the needles. In the case of cones, however, only five extracts were prepared by pooling 4 trees per population due to a lack of material. A total of 64 different extracts were prepared (20 needle bog, 20 needle forest, 9 bark bog, 9 bark forest and 6 cone bog = 64).

### Cell culture and bioactivity assays

PC12-AC cells of the clonal derivate of the rat pheochromocytoma cell line (American Type Culture Collection (ATCC), Manassas, VA, USA) [20] were maintained in 10 cm dishes with 10 mL complete media (RPMI, 10% horse serum, 5% new born calf serum) at 37 °C with 5% atmospheric CO_2_. Complete media provided a normoglucose environment (11 mM glucose). Bioactivity was assessed as previously described [19, 21]. To elicit glucose toxicity (hyperglycemia), complete medium was replaced with this same serum-free media supplemented with 150 mM glucose and 0.025% Bovine Serum Albumin (BSA). Following 96 h (hyperglycemia assays) of treatment, the cell proliferation reagent WST-1 (Roche Diagnostics, Laval, Quebec, Canada) was added to each well and incubated for 60 min before spectrophotometric analysis at 420 nm (formazan) and 620 nm [22]. Cultures were compared to cell-free treatment media incubated for the same period. Cell number per well was calculated from standard curves derived from wells containing known cell densities. Standardization allowed data to be compared across replicates. For LD_50_ assessments, each extract was tested at eight different concentrations in quintuplicate over two separate experiments (*n* = 15 per concentration). For hyperglycemia assays, maximal test concentrations were at least one log concentration below their LD_50_ in normoglucose media. Each extract was tested at four different concentrations in a minimum of triplicate measures (*n* = 3–6). Data from control cultures were combined across plates (*n* = 54). Percent viability was calculated as follows: % viability = cell number _(treatment well)_ / mean cell number_(control)_. Assigned bioactivities (cytotoxic, cytoprotective, or mitogenic) based on this screen were validated by direct assessment of viable cell number using Trypan Blue hemocytometer counts.

### DPPH antioxidant assay

Extracts were tested for their radical scavenging activity using the 1,1-diphenyl-2-picryl-hydrazyle radical (DPPH) test as in [23]. Ascorbic acid (AA) was used as a control to generate a standard curve for the calculation of its IC_50_: the concentration where 50% of the DPPH radical is quenched by AA. The following Least squares regression equation was used [${\hat{y}}_i-{y}_0=\frac{\sum \limits_i\left\{\left({x}_i-{x}_0\right)\left({y}_i-{y}_0\right)\right\}}{\sum \limits_i{\left({x}_i-{x}_0\right)}^2}\left({x}_i-{x}_0\right)$] in order to determine the line of best fit for the IC_50_ calculation where y = absorbance at 517 nm and x = concentration in ppm. This method is employed over the traditional method of discerning the linear portion of the line visually and is a more accurate method of extrapolation. Pure catechin and epicatechin compound was used as positive controls for comparison to crude plant extract samples. Extracts identified as having bioactivity in both screening paradigms and following direct validation were tested to determine whether antioxidant activity correlated with cytoprotection or mitogenicity in hyperglycemic media. Three independent assays were run with samples tested in duplicate.

### Total phenolics test

The Folin-Ciocalteu method was used as previously described [24], and later modified by Harris et al. (2008). A standard curve was constructed using dilutions of quercetin (Sigma Aldrich Co) at concentrations of 1.0, 0.75, 0.5, 0.25, 0.10, and 0.05 mg/mL. Eight hundred micro liters of Folin reagent was added to 160 μL of sample with 540 *μ*L 7.5% NaHCO_3_ and stored in the dark for 2 h. Absorbance was recorded at 725 nm using a spectrophotometer (Molecular Devices SpectraMax 5, Molecular Devices, Sunnyvale, CA, USA). Fifteen milligrams of crude extract was re-solubilized in 80% ethanol at 10 mg/mL inside 1.5 mL centrifuge tubes. The samples were then vortexed until totally dissolved and sonicated for 15 min. These extracts were dyed using the same procedure and the concentration determined using the standard curve.

### Ultra-performance liquid chromatography-quadrupole time-of-flight mass spectrometry (UPLC-Q-TOF)

Samples were prepared by dissolving 40 mg of crude extract in 1 mL of methanol (Sigma Aldrich, St Louis, MO, USA). A standard of 600 μL of resveratrol (1 mg/mL) (Sigma) was added to 500 *μ*L of extract to make a total volume of 1100 *μ*L. The mixture was filtered through a 0.2 μm PTFE nylon membrane filter (Chromatographic Specialties Inc., Brockville, ON, Canada) and sonicated for 5 min before injection.

Analyses were undertaken on an Acquity UPLC coupled with XevoG2 QTOF system (Waters Inc., Milford, MT, USA). UPLC analyses were performed on a Waters Acquity system. Separations were performed on a BEH C18 1.7 μm, 2.1 × 100 mm column (part #186002352; serial #02113226415705, LANSET# General Purpose 2.1 × 100 BEH) connected with a VanGuard pre-column 2.1 × 5 mm. Mobile phases: A, water+ 0.1% formic acid, and B-acetonitrile + 0.1% formic acid (Fisher Optima LC-MS). Flow rate 0.8 ml/min (back pressure at starting conditions = 10,000 PSI). Column temperature, 65 °C, sample temperature 4 °C. Mobile phase composition of B, 0–2 min 2% B isocratic, 2–5 min linear gradient 2–100% B, 5–6 min 100% B isocratic, 6–8 min return to initial mobile phase composition of 2% B isocratic. A 5 μL injection was performed through a 10 μL loop followed by strong wash 200 μL (50% acetonitrile+ 50% water) and weak wash 600 μL (10% acetonitrile + 90% water). Q-TOF was operated in positive and negative electrospray ionization modes. MassLynx software (Version 4.1) was used to acquire high and low energy spectra in MS^e^ ESI+ and MS^e^ ESI- modes within the mass range of 100–1500 Da. Lock mass was set with Leucine Enkephalin C^12^ at 556.2615 Da [M + H]^+ 1^ and 554.261 Da [M-H]^− 1^, source and desolvation temperatures were 150 °C and 500 °C respectively. Cone gas and desolvation gas (nitrogen) were set at 50 and 1200 L/hr). The identification of the compounds is based on matching the molecular ion acquired in low fragmentation setting and the major fragments acquired in high fragmentation setting within the mass accuracy of 5 PPM.

## Results

### Percentage yield

Upon extraction, 18, 25 and 30% of dried needles, bark and cone were sequestered.

### Bioactivity

**Fig. 2:**
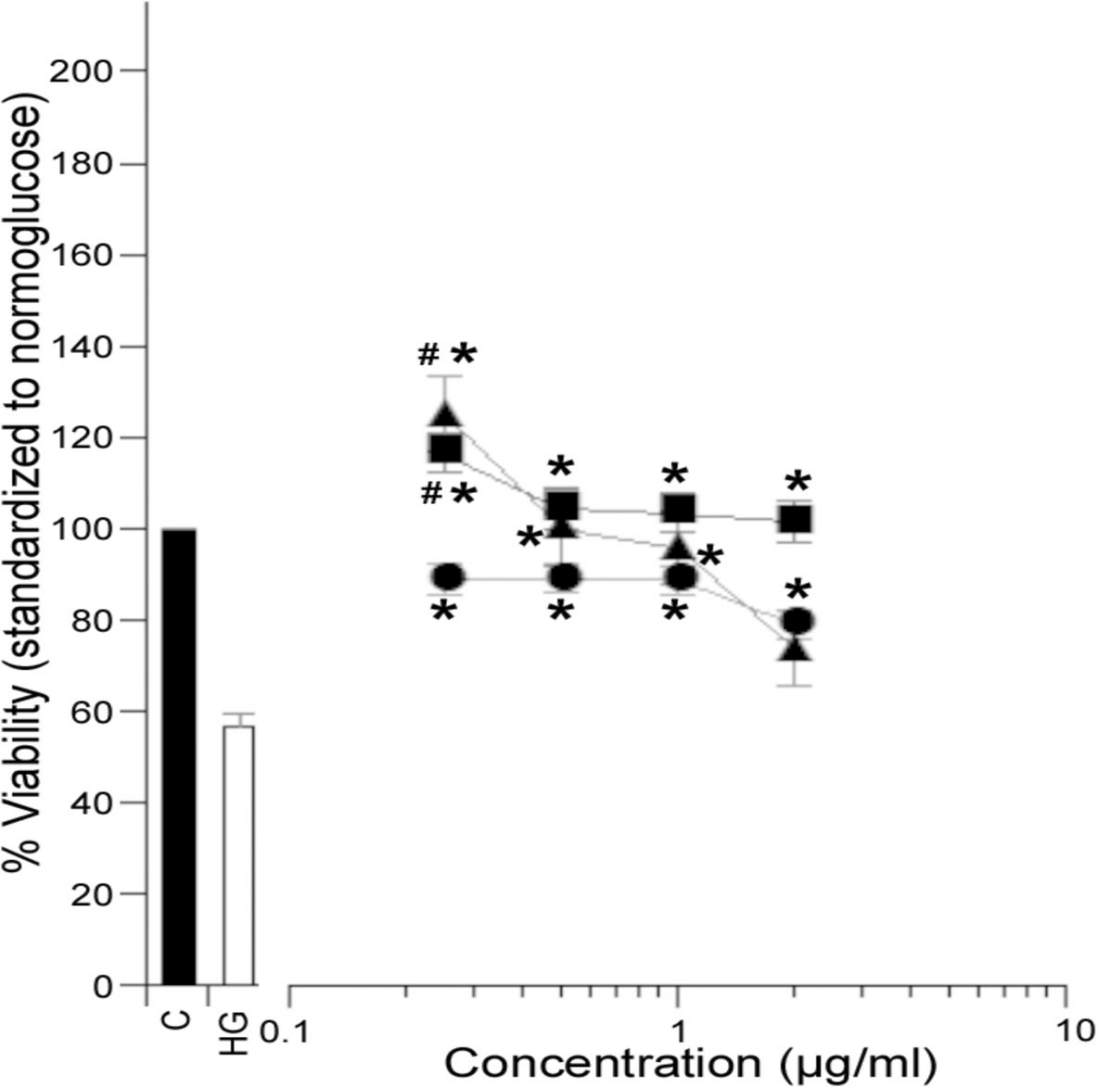
Comparison of cytoprotective and mitogenic activities of *P. mariana* needle (●), bark (■) and cone (▲) extracts in PC12-AC grown in hyperglycemic media. MANOVA analysis was used to average all extracts based on their organ type and concentration. Significant cytoprotection (_*_) or mitogenic activity (#) was afforded when Dunnett post hoc tests were *p* ≤ 0.05 as compared to the normoglucose **(C)** and high glucose **(HG)** controls. All organs showed significant protection at all concentrations except cone at 2 μg/mL (needle *n* = 120, bark *n* = 60, cone *n* = 18)

**Fig. 3:**
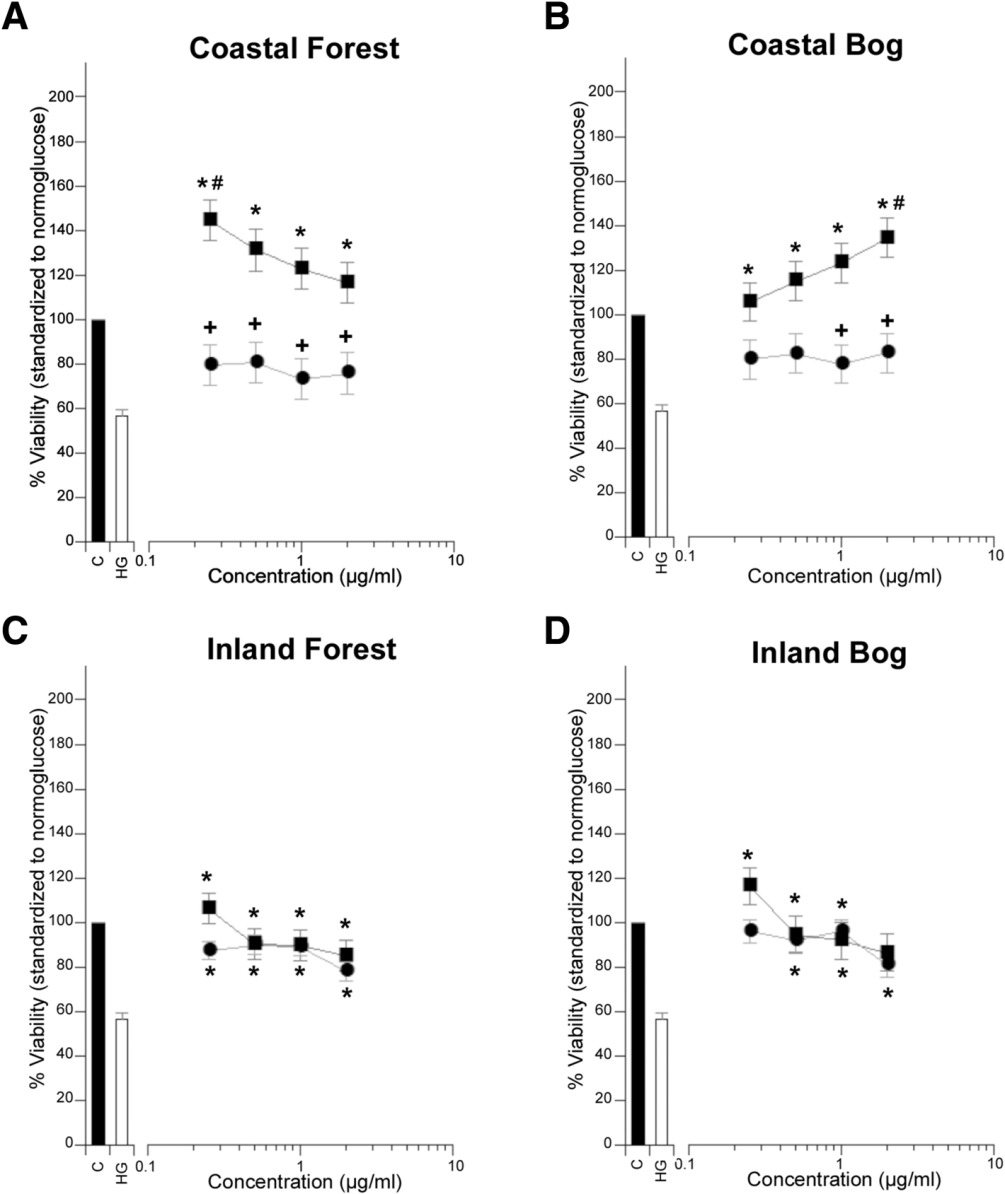
Comparison of cytoprotective and mitogenic activities of *P. mariana* extracts on PC12-AC by growth environment. MANOVA analysis was used to determine the mean viability with respect to habitat (forest/ bog), growth environment (coastal/inland), organ type (Needle = ●, Bark = ■) and concentration (0.25, 0.50, 1.00, 2.00 *μ*g/mL) interactions. Significant protection (_*_) or mitogenic activity (#) was identified by Dunnett post hoc tests (*p* ≤ 0.05) by comparison to the (**C**) normoglucose or (**HG**) high glucose controls respectively. The same *p* value was used to denote a significant difference between needle and bark (**+**) at specified concentrations. **a** Comparison of bark and needle extract protection in coastal forest environments (bark *n* = 12, needle *n* = 12). **b** Comparison of bark and needle extract protection in coastal bog environments (bark *n* = 12, needle *n* = 12). **c** Comparison of bark and needle extract protection in inland forest environments (bark *n* = 18, needle *n* = 51). **d** Comparison of bark and needle extract protection in inland bog environments (bark *n* = 18, needle *n* = 45)

LD_50_ values for all extracts were 25 μg/mL or higher. Consequently, each extract was screened at 0.25, 0.5, 1 and 2 *μ*g/mL for capacity to alter glucotoxicity in vitro (Tables 1, 2, 3, Figs. 2, 3, Additional files 1-3: Figures S1-S3). Maximal concentrations of extracts were at least one log concentration below their LD_50_ in normoglucose media. Glucotoxicity (high glucose, HG) reduced the number of viable cells to 56.8% that of normoglucose controls (C) (Figs. 2 and 3). Samples were stated as biologically active if at least two or more concentrations elicited a statistically significant change in viable cell number. Compounds that increased viable cell number to values significantly higher than the normoglucose controls (> 100%) were considered mitogenic. Compounds that significantly protected cells from high glucose toxicity (56.8%) without exceeding normoglucose viability values were considered cytoprotective; compounds that enhanced glucotoxicity at any concentration were considered cytotoxic (Tables 1, 2, 3).

**Table 1:** Bioactivity of *Picea mariana* needle extracts in PC12-AC grown in hyperglycemic media

Extract ID^a^	Bioactivity in hyperglycemic media^b^
1FN	Cytoprotective
1BN	None
2FN	None
2BN	None
3FN	None
3BN	None
4FN	None
4BN	None
5FN	None
5BN	None
6FN	Cytoprotective
6BN	Cytoprotective
7FN	None
7BN	Cytoprotective
8FN	Mitogenic
8BN	Mitogenic
9FN	None
9BN	Cytoprotective
10FN	Cytoprotective
10BN	None
11FN	None
11BN	None
13FN	None
13BN	None
14FN	None
14BN	None
15FN	None
15BN	None
16FN	Cytoprotective
16BN	None
17FN	Cytoprotective
17BN	Cytoprotective
18FN	Cytoprotective
18BN	Cytoprotective
19FN	None
19BN	Cytoprotective
20FN	None
20BN	None
21FN	Cytoprotective
21BN	None

^a^The extract ID code defines the growth environment (Area#1–21, Fig. 1), habitat (*B* Bog or *F* forest) and organ (*N* needle). For example, 1FN is an extract prepared from needles collected in a forest habitat at location #1. Populations are listed in ascending order from coastal west (1) to inland east (21) with forest (F) and bog (B) populations next to one another for comparison

^b^Bioactivity was classified comparing a 96 h treatment in hyperglycemic (150 mM) serum-free media. Viable cell number following extract treatment was established using the WST-1 assay compared to standard curves of known cell number. Vehicle control was 0.1% DMSO. Extracts that at two or more concentrations increased viable cell number to values significantly higher than the normoglucose controls (> 100%) were classified as mitogenic, those which significantly protected cells from high glucose toxicity without apparent mitogenic activity were classified as cytoprotective, and those which enhanced glucotoxicity were classified as cytotoxic. Statistics were ANOVA, *post-hoc* Tukey tested vs. vehicle-treated cultures in normo- or high glucose media. All concentration-response data and statistical analyses are presented in Additional file 1: Figure S1

**Table 2:** Bioactivity of *Picea mariana* bark extracts in PC12-AC grown in hyperglycemic media

Extract ID^a^	Bioactivity in hyperglycemic media^b^
1FB	Mitogenic
1BB	Mitogenic
2FB	Cytoprotective
2BB	Cytoprotective
3FB	Cytoprotective
3BB	Cytoprotective
4FB	Cytoprotective
4BB	Cytoprotective
5FB	None
5BB	None
6FB	Cytoprotective/Mitogenic
6BB	Cytoprotective/Mitogenic
7FB	Cytoprotective
7BB	Cytoprotective
11FB	None
11BB	Cytoprotective
14FB	None
14BB	None

^a^The extract ID code defines the growth environment (Area#1–21, Fig. 1), habitat (*B* Bog or *F* forest) and organ (*B* bark). For example, 1FB is an extract prepared from bark collected in a forest habitat at location #1

^b^Bioactivity was classified using a 96 h treatment in hyperglycemic (150 mM) serum-free media. Viable PC12-AC cell number following extract treatment was established using the WST-1 assay compared to standard curves of known cell number. Vehicle control was 0.1% DMSO. Extracts that at two or more concentrations increased viable cell number to values significantly higher than the normoglucose controls (> 100%) were classified as mitogenic, those which significantly protected cells from high glucose toxicity without apparent mitogenic activity were classified as cytoprotective, and those which enhanced glucotoxicity were classified as cytotoxic. Samples classified with dual bioactivities (ie. Cytoprotective/Mitogenic) are possible when two concentrations satisfy our definition of one bioactivity (ie. Cytoprotective) and two other concentrations from the same sample are in another category (ie. Mitogenic). Statistics were ANOVA, *post-hoc* Tukey tested vs. vehicle-treated cultures in normo- or high glucose media. All concentration-response data and statistical analyses are presented in Additional file 2: Figure S2

**Table 3:** Bioactivity of *Picea mariana* cone extracts in PC12-AC grown in hyperglycemic media

Extract ID^a^	Bioactivity in hyperglycemic media^b^
3BC	Cytoprotective
7BC	Cytoprotective
11BC	Mitogenic
12BC	Mitogenic
14BC	None
15BC	None

^a^The extract ID code defines the growth environment (Area#1–21, Fig. 1), habitat (*B* Bog or *F* forest) and organ (*C* cone). For example, 1BC is an extract prepared from cones collected in a bog habitat at location #1

^b^Bioactivity was classified comparing a 96 h treatment in hyperglycemic (150 mM) serum-free media. Viable cell number following extract treatment was established using the WST-1 assay compared to standard curves of known cell number. Vehicle control was 0.1% DMSO. Extracts that at two or more concentrations increased viable cell number to values significantly higher than the normoglucose controls (> 100%) were classified as mitogenic, those which significantly protected cells from high glucose toxicity without apparent mitogenic activity were classified as cytoprotective, and those which enhanced glucotoxicity were classified as cytotoxic. Statistics were ANOVA, *post-hoc* Tukey tested vs. vehicle-treated cultures in normo- or high glucose media. All concentration-response data and statistical analyses are presented in Additional file 3: Figure S3

We found that 50% of all organ extracts improved viability of PC12 cultures under hyperglycemic conditions (Tables 1, 2, 3, Additional files 1-3: Figures S1-S3). Moreover, all extracts were well-tolerated in that none of them enhanced glucose cytotoxicity. We identified 24 cytoprotective (38% of total) and seven mitogenic (9% of total) extracts. Two extracts (3% of total) exhibited both concentration-dependent cytoprotective and mitogenic activities. When comparing the overall bioactivity of extracts prepared from needle, bark and cone collected in all regions and habitats, we found that protective effects were maximal at the lowest tested concentrations in all cases and were not organ-specific with the exception of bark and cone which were more effective mitogens in high glucose media than needle extracts (Tables 1, 2, 3, Additional files 1-3: Figures S1-S3, Fig. 2).

### Regional variation

A primary concern of the TAAM team is to provide laboratory data assessing the importance of plant selection and regional variation emphasized by the elders and healer of the Eeyou Istchee in their traditional preparations. Their insights are crucial to ensuring that plant usage will yield similar benefits in all communities. We found that 50 % of *P. mariana* needle, bark, and cone extracts (32 extracts) did not exhibit significant biological activity in our screening paradigm. To test whether extract efficacy depended upon the growth habitat where the plant had been harvested, we compared efficacy of needle and bark extracts collected in bog and forest habitats. Cone extracts were not included in this analysis as we were only able to collect cones from bog sites.

We did not detect any statistically significant differences between the bioactivities of needle and bark extracts grown in different habitats (data not shown). To test whether growth environment and habitat interacted to produce organs with different bioactivities, samples collected in different growth habitats were grouped into coastal or inland environments (Fig. 1). This analysis revealed a statistically significant interaction among growth environment, habitat, and organ bioactivity (Fig. 3). Extracts prepared from bark collected in coastal environments showed cytoprotection where needles extracts did not (Fig. 3a and b). Moreover, in coastal-derived extracts, activity was maximal (and concentration-dependent) at low concentrations in extracts prepared from bark collected in a forest habitat (Fig. 3a) and at high concentrations in extracts prepared from bark collected in a bog habitat (Fig. 3b). At their highest percent of cell viability, extracts were even mitogenic. This interaction was organ-specific. Neither growth environment nor habitat altered the cytoprotective efficacy of needle extracts (Fig. 3a-d).

### Total phenolics and antioxidant activity

**Fig. 4:**
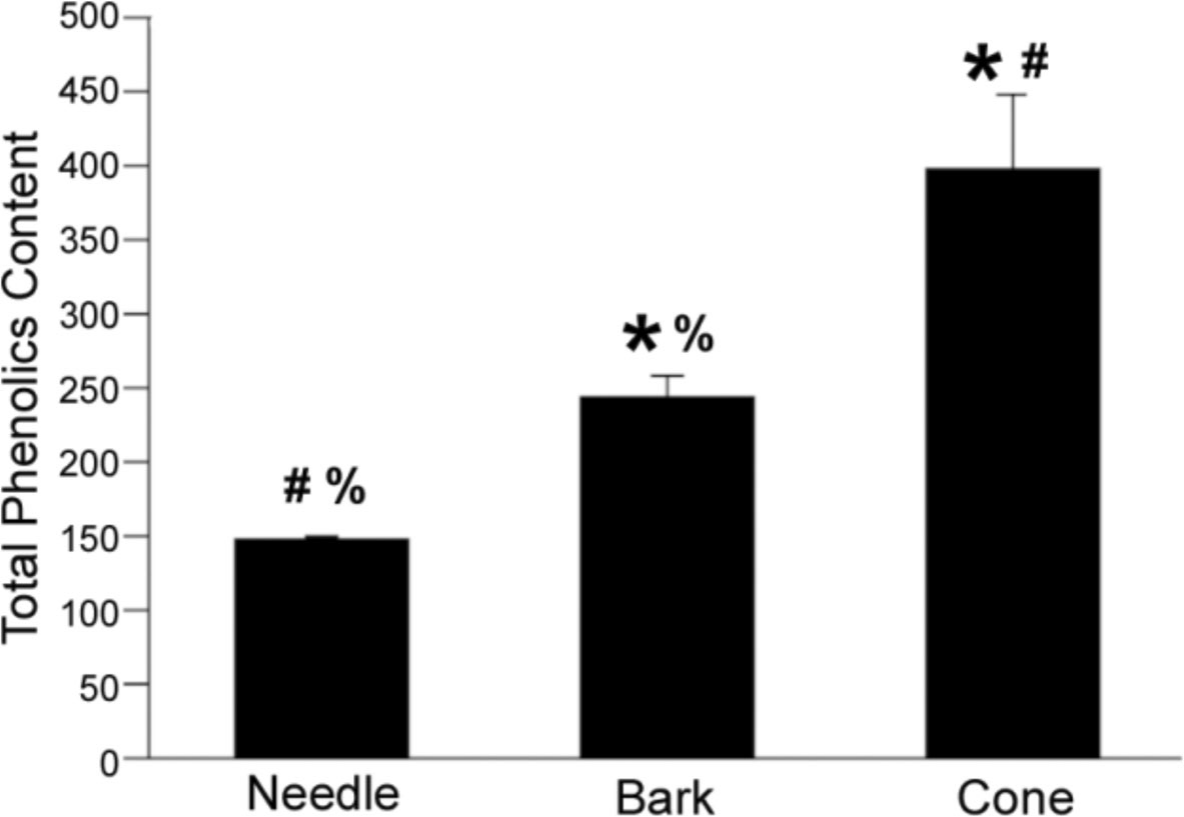
Total phenolic content in terms of quercetin equivalents, of *P. mariana* needle, bark and cone extracts. Needle, bark and cone phenolic content was found to be significantly different from each other at *p* ≤ 0.05 ((_*_) = significantly different from needle; (**#**) = significantly different from bark; (**%**) = significantly different from cone). Cone had the highest phenolic content with 398.68 (SE = 48.77, *n* = 5), followed by bark at 244.08 (SE = 14.51, *n* = 18) and needle with 146.34 *μ*g/mg extract (SE = 3.78, *n* = 40)

These data suggest that the amounts of biologically active compounds are responsible for organ-specific, habitat-dependent cytoprotective bioactivities of the extracts. It has previously been suggested [17] that antioxidant activity is responsible for antidiabetic activities in *P. mariana* cone extracts. To further explore this hypothesis, we measured total phenolic content in the needle, bark and cone extracts (Fig. 4) and correlated this content with the antioxidant activity of selected extracts using the Folin-Ciocalteu total phenolics method and DPPH antioxidant tests employed in previous TAAM studies [17].

We found that the combined phenolic content was highest in cone extracts and lowest in needle. For each milligram of extract, 146.5 ± 4.2 μg (needles), 270.3 ± 13.9 *μ*g (bark) and 398.7 ± 48.8 l *μ*g (cone) were phenolic compounds (Fig. 4).

**Fig. 5:**
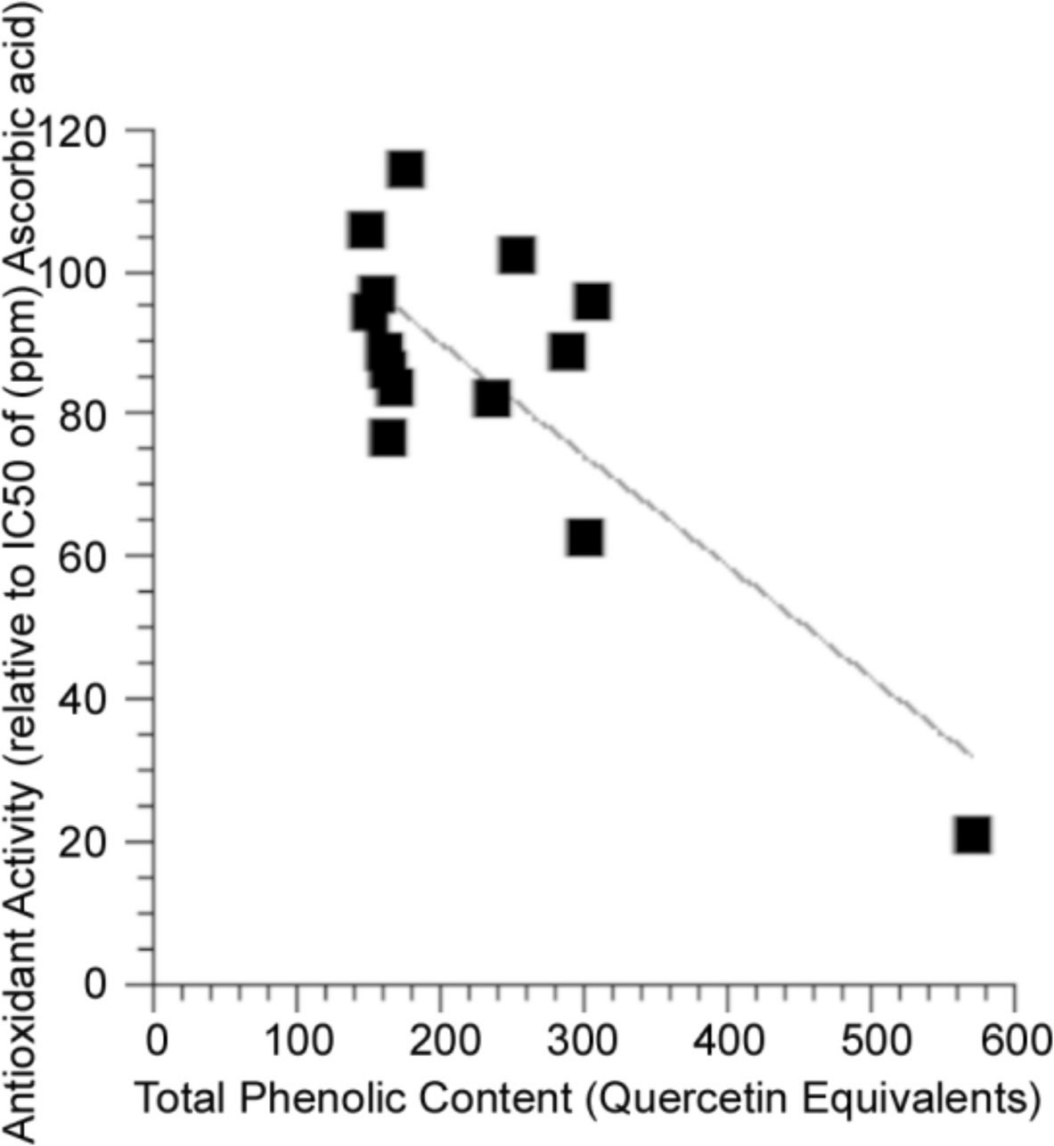
Regression analysis of total phenolic content of *P. mariana* extracts with respect to antioxidant activity. Total phenolic content is measured in terms of quercetin equivalents and antioxidant activity is calculated with respect to the ascorbic acid standard curve. The trend shows that as phenolic content increases the amount of plant extract (EC_50_ in ppm) necessary to reach the IC_50_ of ascorbic acid decreases and therefore, antioxidant activity increases with increasing phenolic content (*r*^2^ = 0.63, *p* ≤ 0.05, *n* = 14). The data passed a normality test in order to ensure that it follows a normal distribution

PH antioxidant assays were completed on 14 selected extracts and plotted with respect to phenolic content (Fig. 5). A significant correlation between antioxidant activity and total phenolic content was detected (*r*^2^ = 0.63, *p* < 0.05, Fig. 5).

**Fig. 6:**
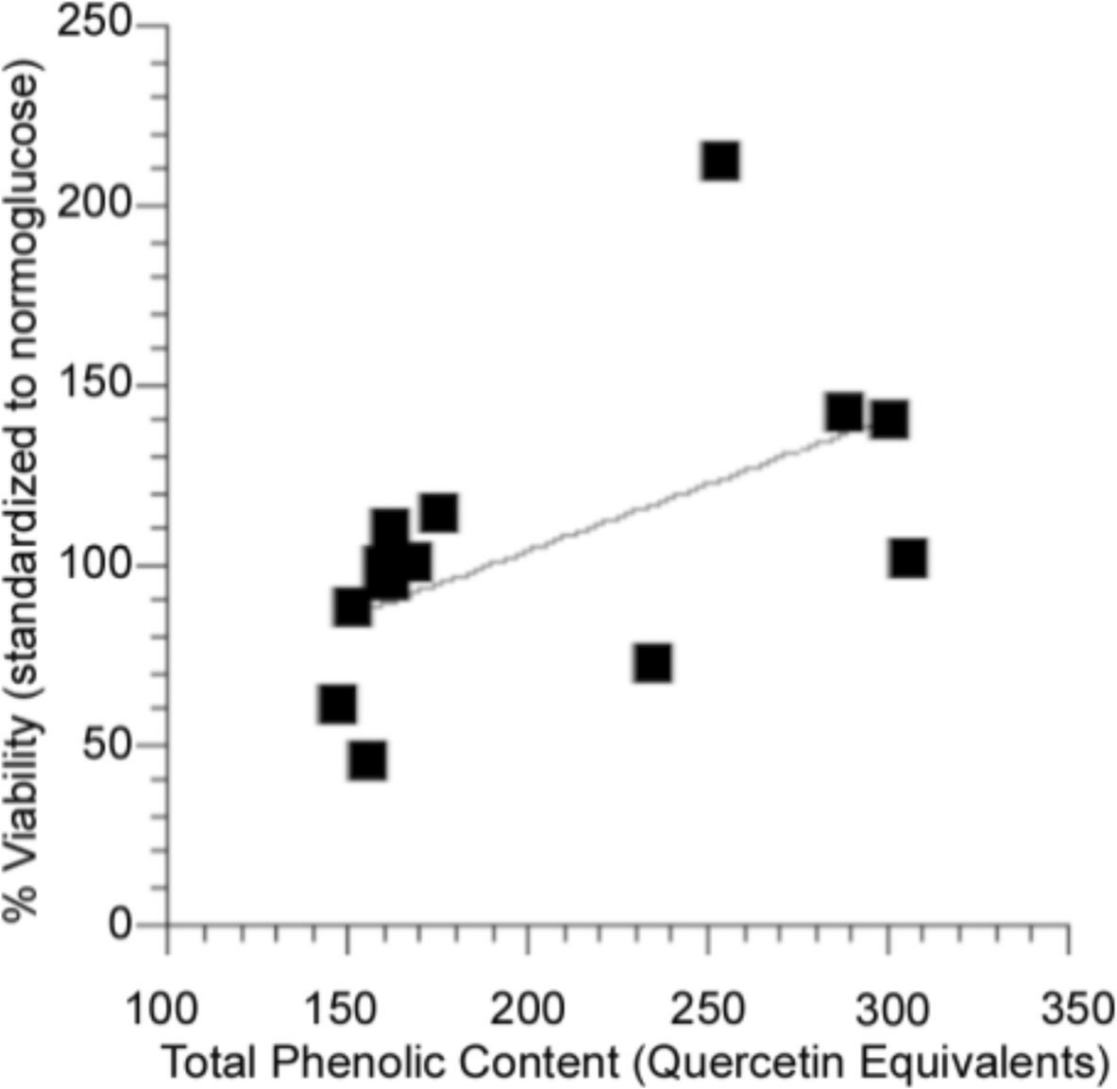
Regression analysis of total phenolic content of of *P. mariana* extracts with respect to bioactivity in PC12-AC in high glucose media. The trend shows that as phenolic content increases the percent viability increases (*p* ≤ 0.05, *n* = 13)

**Fig. 7:**
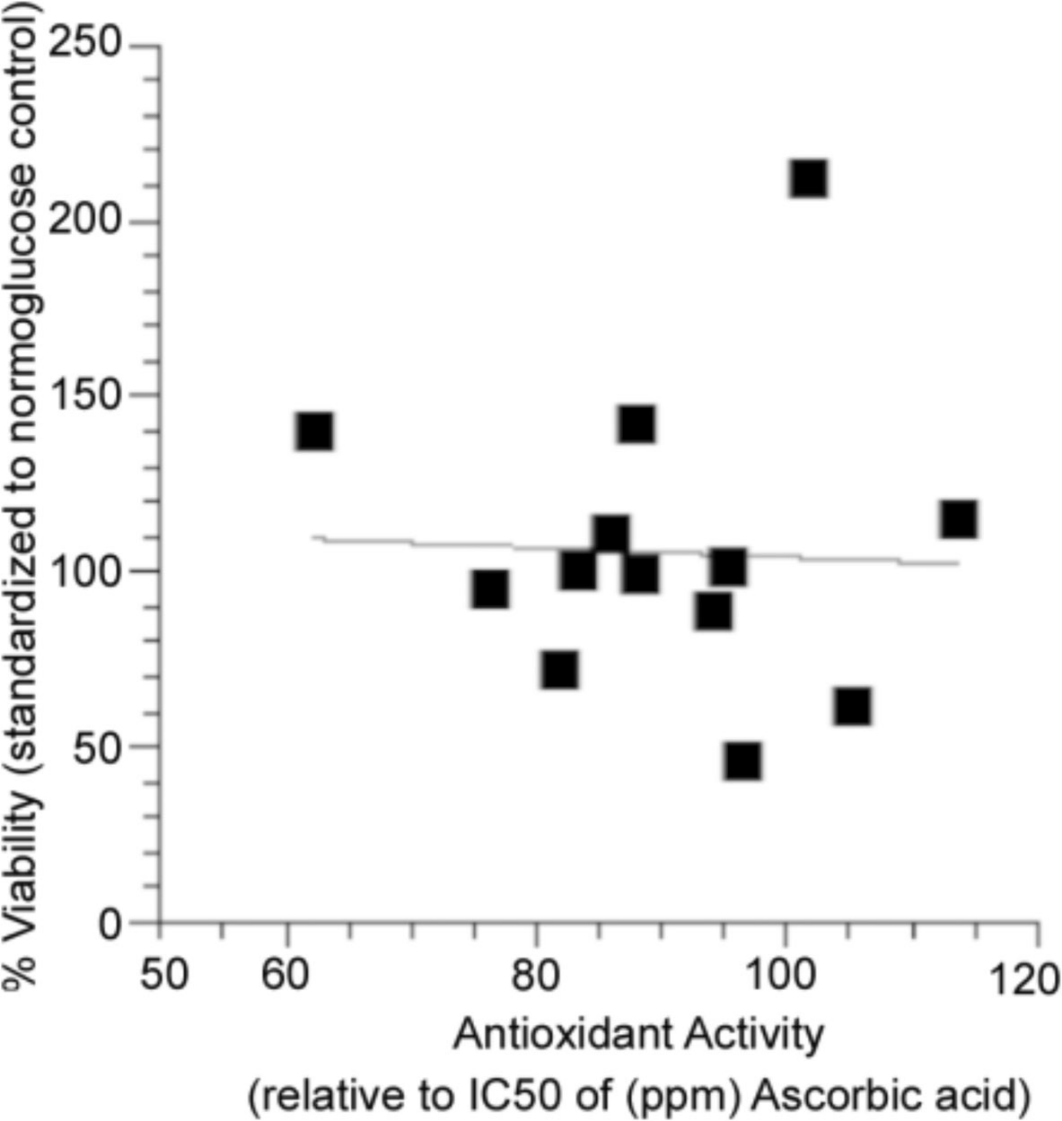
Regression analysis of antioxidant activity of *P. mariana* extracts with respect to PC12-AC bioactivity in high glucose media. Antioxidant activity is measured with respect to ascorbic acid and bioactivity is represented as percent viability with respect to normoglucose control. We do not see any trend; therefore, the antioxidant activity is not likely the main mechanism of protection (*p* = 0.89, *n* = 13)

Total phenolic content positively correlated with bioactivity (*r*^2^ = 0.30, *p* = 0.05, Fig. 6, Tables 1, 2, 3). Antioxidant activity, however, was not correlated with bioactivity (*r*^2^ = 0.046, *p* = 0.460, Fig. 7). Taken together, these results suggest that, while the phenolic content of organ extracts prepared from plants harvested in different habitats and growth environments likely underlies bioactivity, the oxyradical scavenging capacity of these phenolics is not the primary mechanisms of action.

### UPLC-QTOF analyses

Analyses of the methanol extracts of needle, bark and cone extracts were completed by Dr. Ammar Saleem at the LANSET core facility, University of Ottawa and yielded the identification of two classes of compounds – phenolics (Additional file 4: Figure S4-a) and terpenes (Additional file 4: Figure S4-b). Using the natural products data base that we have created, a total of 32 phenolic and terpene compounds were detected in *P mariana* needle, bark and cone extracts (Table 4, Additional file 5: Figure S5a-c). All plant parts contained proanthocyanidins b6 and b3, as well as abietic acid, dehyroabietic acid and oxodehydroabietic acid. Eleven compounds were confirmed as present in needles, with an additional 10 tentatively compounds and four in trace quantities. Kaemferol-7-O- glucoside was unique to the needles. The bark contained 8 confirmed compounds and 6 tentatively present and 2 in trace quantities with picealactone b as a unique component. The cones have not been previously analysed and revealed 10 confirmed compounds, 7 tentatively present and 5 trace compounds. Two significant compounds here are resveratrol, with antioxidant, antidiabetic and other biological activities [25], as well as taxifolin, also an antioxidant but structurally related to quercetin, which has previously been reported to protect against oxidative stress induced apoptosis [26]. Additional information on the new UNIFI based identification system used here and based on diagnostic low and high energy spectra will be published elsewhere.

**Table 4:** List of compounds in *P. mariana* needle, bark and cone extracts identified by ESI-QTOF positive electrospray ionization. C (Retention time match ±0.02 min, low and high energy spectra match within ±5 ppm mass accuracy); P (low and high energy spectra match within 5 ppm mass accuracy); T (signal intensity of quasi molecular ion below 1500 counts); A (absent)

#	Compound	Molecular formula	Accurate mass	[M + H]^+ 1^	Rt. (min)	Needle	Bark	Cone
Phenolics								
1	3′,4′-Dihydroxyacetophenone	C8H8O3	152.0473	153.0553	2.71	C	C	A
2	4-Hydroxybenzoic acid	C7H6O3	138.0317	139.0396	2.66	C	T	T
3	4-Methylcatechol	C7H8O2	124.0524	125.0604	2.98	T	T	T
4	Coumarin	C9H6O2	146.0368	147.0447	2.95	C	C	A
5	Dihydroquercetin	C15H12O7	304.0583	305.0662	3.00	A	T	C
6	Isorhapontigenin	C15H14O4	258.0892	259.0971	3.11	T	A	C
7	Isorhapontigenin 3-O-beta-D-glucopyranoside	C21H24O9	420.1420	421.1500	3.12	A	A	P
8	Kaempferol	C15H10O6	286.0477	287.0557	3.08	A	A	C
9	Kaempferol 3-(3″-p-coumaryl-6″-ferulylglucoside)	C40H34O16	770.1847	771.1926	3.74	P	A	A
10	Kaempferol-7-O-glucoside	C21H20O11	448.1006	449.1085	2.94	C	A	A
11	Piceatannol	C14H12O4	244.0736	245.0815	2.91	C	A	C
12	Procyanidin B3	C30H26O12	578.1424	579.1504	3.92	C	C	C
13	Procyanidin B6	C30H26O12	578.1424	579.1504	2.65	C	C	C
14	Resveratrol	C14H12O3	228.0786	229.0866	3.04	T	A	C
Terpenes								
15	Picealactone C	C20H22O4	326.1518	327.1597	3.58	A	P	A
16	Isolariciresinol	C20H24O6	360.1573	361.1652	3.70	P	A	A
17	Picealactone A	C20H22O3	310.1569	311.1648	4.48	A	P	T
18	Dehydroabietic acid	C20H28O2	300.2089	301.2169	3.91	C	C	C
19	12-Hydroxydehydroabietic acid	C20H28O3	316.2038	317.2118	4.37	P	P	P
20	Abieta-8,11,13-trien-7-one	C20H28O	284.2140	285.2219	4.19	A	P	P
21	7-Oxodehydroabietic acid	C20H26O3	314.1882	315.1961	4.42	C	C	C
22	Abietic acid	C20H30O2	302.2246	303.2325	4.97	C	C	C
23	Sandaracopimaric acid	C20H30O2	302.2246	303.2325	4.52	C	C	A
24	Typhasterol	C28H48O4	448.3553	449.3632	5.20	P	A	A
25	(+)-Picealactone B	C20H22O4	326.1518	327.1597	3.15	P	A	A
26	(+)-Lariciresinol	C20H24O6	360.1573	361.1652	3.73	P	A	A
27	Gibberellin A1	C19H24O6	348.1573	349.1652	4.63	P	A	T
28	Gibberellin A15	C20H26O4	330.1831	331.1910	3.83	P	P	P
29	Gibberellin A51	C19H24O5	332.1624	333.1703	4.01	P	P	P
30	Gibberellin A29	C19H24O6	348.1573	349.1652	4.66	P	A	T
31	Gibberellin A7	C19H22O5	330.1467	331.1546	3.10	T	A	P
32	Neocembrene	C20H32	272.2504	273.2583	5.12	A	A	P

### Bioactivity of taxifolin

To investigate whether taxifolin elicits cytoprotective effects and may contribute to the observed activity of cone extracts, a pure taxifolin standard (Sigma Aldrich, Oakville, ON) was administered at increasing non-toxic concentrations (0.8, 1.6 and 3.2 μM) to PC12 cells exposed to glucotoxicity (Additional file 6: Figure S6). Cytoprotection was significant at 1.6 μM with nearly identical concentration-dependent response as seen for quercetin [22].

## Discussion

There is a paucity of strategies effective in managing chronic pain, infection, paralysis, and loss of sensation in association with peripheral diabetic neuropathy [8, 27]. Based on our ethnobotanical studies, we have observed that preparations from various organs of *P. mariana* are used to treat slow healing infections, sores and numbness (particularly of the extremities), all symptoms related to diabetic neuropathy. We demonstrate for the first time that *P. mariana* needles and bark are effective protectors of peripheral neuronal precursor survival when exposed to hyperglycemic conditions. We confirm the protection of cones, which was first demonstrated by our team [17]. We have also shown that harvest location can impact the cytoprotective activities of *P. mariana*, particularly for bark extracts, which tended to be more effective when obtained from inland forest populations than from coastal and inland bog populations. In a similar fashion, [28, 29] showed a latitudinal trend in key phenolic concentration but failed to find a correlation with bioactivity. In general, the strength of the observed activity of needle, bark and cone is correlated with the total phenolic concentration of the extracts. The one notable exception was a cone extract that was suspected to have caused oxidation, a negative response, in PC12-AC cells. The presence of too many antioxidants can actually be harmful, resulting in oxidation and therefore a decrease in cell survival. This situation was observed in preliminary DPPH tests at higher extract concentrations [30].

These in vitro findings validate the importance of *P. mariana’*s role as part of the Cree pharmacopoeia and underline the importance of plant selection and extract strength. In a review of Canadian aboriginal plant use, [31], it has been reported that this plant is used by the Cree to treat many ailments including heart problems, high blood pressure as well as many infectious and inflammatory conditions. To TAAM’s ethnobotanical and in vitro findings [14, 15, 17, 32], we have contributed additional positive evidence that boreal plants from the Cree pharmacopoeia are capable of treating diabetes neuropathy symptoms. Specifically, we have shown at the cellular level, that this traditional medicine may be effective in the treatment of this “new” disease afflicting First Nation peoples, namely the potential to manage diabetic peripheral symptoms by protecting peripheral neurons and their precursors from hyperglycemic insult.

These results are similar to those found in Harris et al. (2008) for *P. glauca,* except that all *P. mariana* organs showed protection. In contrast to Harris et al. (2008) findings, *P. mariana* needles were generally the lowest protector against diabetic insult. One factor that should be re-examined in the case of *P. glauca* is concentration range. It may be necessary to test bark and cone at much lower concentrations than 10 μg/mL. This is recommended because our graphs often showed a decreasing dose response from the lowest concentration to the highest (Fig. 3). Conversely, given strong correlations in both studies with total phenolic content, these data could also suggest species differences in the phenolic constituents of the different organs that underlie the cytoprotective and mitogenic bioactivities. This is a good argument for the comprehensive fractionation-based assays of organs collected at different locations and under different growth habitats in order to identify active components.

We further show that extract efficacy depends upon careful selection of plant organs depending upon growth environment and habitat as indicated by the Cree elders and healers of Eeyou Istchee. This is an important finding as it supports the efforts of traditional practitioners in ensuring equivalent benefits across communities. Variation in activity by growth environment was observable mainly for bark. In this case, we recommend inland populations or, if harvesting in proximity to the coast, to choose forest sites instead of bog sites. Conveniently, this is easier ground to navigate and a less fragile habitat. However, if a bog is the only site available for harvesting, it may be possible to achieve the same level of protection in coastal sites, for example, by simply using more plant material per treatment preparation. In the case of needles, neither growth environment nor habitat altered the cytoprotective efficacy of extracts, so no special recommendations are warranted. A lack of specificity should allow collectors to avoid overharvesting in one area and, therefore, decreases the possibility of harmful environmental impacts.

Using UPLC-QTOF method, a total of 14 phenolics and 18 terpenes were detected or tentatively identified in needle, bark and cones extracts. Among these phenolics, piceatannol was detected in cone and needle extracts, yet absent from bark extract. On the other hand, the identities of resveratrol and isorhapontigenin were only confirmed in the cone extract. These three compounds belong to a group of compounds called stilbenes and have shown a wide range of biological activities such as antidiabetic, antioxidant, anti-aging, anti-cancer and anti-inflammatory activities [33–36]. Of note, resveratrol, is the most studied stilbene and is a known cytoprotector found in red wine [37] while isorhapontigenin is a novel stilbene that was recently identified in red wine. Stilbenes were previously detected in a number of unrelated gymnosperm and angiosperm species such as *Abies*, *Picea*, *Pinus*, *Juniperus*, *Rheum* and *Morus* species [38]. Besides the properties or activities listed above, stilbenes have been shown to improve blood circulation and have antimicrobial activities [38, 39]. Another interesting phenolic group is the proanthocyanidins; they constitute a subclass of flavonoids that has attracted growing attention due to their potential health promoting effects [40]. Procyanidin B3 and Procyanidin B6 are detected in needle, bark and needle extracts. On the other hand, the flavanonols dihydroquercetin or taxifolin was only confirmed in the cone extract but may nonetheless contribute to cytoprotective effects elicited by these extracts (Additional file 5: Figure S5). Concerning terpenes, the conifer biomarkers abietic and dehydroabietic acids as well as 7-Oxodehydroabietic acid were detected in the extracts of the three plant parts while sandaracopimaric acid was only identified in the needle extract. Importantly, abietic acid and dehydroabietic acid were reported to exhibit a potent inflammatory activity and have antioxidant as well as antiobesity and regulation of glucose metabolism [41]. While still preliminary, this approach can identify the bioactive compounds that underlie species differences as well as reflect the observed growth environment and habitat impact upon extract efficiency.

## Conclusion

*P. mariana* is an effective protector of peripheral neuronal precursor survival when cells are subjected to high glucose conditions. All organs tested in this diabetic neuropathy model (needles, bark and cone) proved to be efficacious. As all organ parts are protective and the boreal forest of Canada is highly populated by this tree species, we believe *P. mariana* is an excellent candidate for a renewable source of medicine. Further in vitro investigations are necessary to elucidate the underlying mechanisms of action and phenolic variation. In vivo tests using diabetes specific animal models are also needed to confirm that bioactivity remains upon ingestion. Of course, the successful application of this study’s findings will also depend on the continuation of an already interdependent relationship developed by the TAAM team, health care professionals and the Eeyou Istchee communities. As the bonds we have built are strong, we are confident that not only *P. mariana* but many of the plants from the Cree pharmacopeia will become part of a new holistic form of diabetes treatment, culturally acceptable to the Cree of Eeyou Istchee and supported by traditional practice and evidence-based inquiry.

## Additional files

Additional file 1: **Figure S1.** (A-T). Comparison of cytoprotective, mitogenic and cytotoxic activities of *P. mariana* pooled needle extracts from forest and bog populations in a PC12-AC model of high glucose stress. Populations are listed in ascending order from coastal west [1] to inland east [21] with forest (F) and bog (B) populations next to one another for comparison. Organ type is specified as the last number in the code; needle (N). Therefore, each graph has a code identifying population number, habitat type and organ type above it in that order. Bioactivity was assessed using the formazan dye WST which measures mitochondrial dehydrogenase activity. Treatment wells containing extract were standardized to the normoglucose control (C) and compared to this and the high glucose control (HG) for the determination of protective, mitogenic or toxic effects. A students t-test was used in order to determine the significant difference between the normoglucose control (100%) and the high glucose control (56.799%, *n* = 54 wells/condition, bar graph, *p* ≤ 0.05). Anova analysis was employed in order to compare the % viability for each concentration 0.25, 0.50, 1.00 and 2.00 μg/mL to the normoglucose and high glucose controls. Differences were deemed significant (*cytoprotection, #mitogenic) when *p* ≤ 0.05 (*n* = 3 treatment wells/concentration). (PDF 973 kb)

Additional file 2: **Figure S2.** (A-I). Comparison of cytoprotective, mitogenic and cytotoxic activities of *P. mariana* pooled bark extracts from forest and bog populations in a PC12-AC model of high glucose stress. Populations are listed in ascending order from coastal west [1] to inland east [14] with forest (F) and bog (B) populations next to one another for comparison. Organ type is specified as the last number in the code; bark (B). Therefore each graph has a code identifying population number, habitat type and organ type above it in that order. Bioactivity was assessed using the formazan dye WST which measures mitochondrial dehydrogenase activity. Treatment wells containing extract were standardized to the normoglucose control (C) and compared to this and the high glucose control (HG) for the determination of protective, mitogenic or toxic effects. A students t-test was used in order to determine the significant difference between the normoglucose control (100%) and the high glucose control (56.799%, *n* = 54 wells/condition, bar graph, *p* ≤ 0.05). Anova analysis was employed in order to compare the % viability for each concentration 0.25, 0.50, 1.00 and 2.00 μg/mL to the normoglucose and high glucose controls. Differences were deemed significant (*cytoprotection, #mitogenic) when p ≤ 0.05 (*n* = 3 treatment wells/concentration). (PDF 476 kb)

Additional file 3: **Figure S3.** (A-F). Comparison of cytoprotective, mitogenic and cytotoxic activities of *P. mariana* pooled cone extracts from bog populations in a PC12-AC model of high glucose stress. Populations are listed in ascending order from coastal west [3] to inland east [15] Habitat is the second letter in the code (bog = B) while Organ type is specified as the last number in the code (cone = C). Therefore each graph has a code identifying population number, habitat type and organ type above it in that order. Bioactivity was assessed using the formazan dye WST which measures mitochondrial dehydrogenase activity. Treatment wells containing extract were standardized to the normoglucose control (C) and compared to this and the high glucose control (HG) for the determination of protective, mitogenic or toxic effects. A students t-test was used in order to determine the significant difference between the normoglucose control (100%) and the high glucose control (56.799%, *n* = 54 wells/condition, bar graph, *p* ≤ 0.05). Anova analysis was employed in order to compare the % viability for each concentration 0.25, 0.50, 1.00 and 2.00 μg/mL to the normoglucose and high glucose controls. Differences were deemed significant (*cytoprotection, #mitogenic) when *p* ≤ 0.05 (*n* = 3 treatment wells/concentration). (PDF 171 kb)

Additional file 4: **Figure S4.** (A-B). Chemical structures of compounds in *P. mariana* needles, bark and cone extracts identified by ESI-QTOF positive electrospray ionization. (A) Phenolics, (B) Terpenes. 1: 3′,4′-Dihydroxyacetophenone, 2: 4-Hydroxybenzoic acid, 3: 4-Methylcatechol, 4: Coumarin, 5: Dihydroquercetin, 6: Isorhapontigenin, 7: Isorhapontigenin 3-O-beta-D-glucopyranoside, 8: Kaempferol, 9: Kaempferol 3-(3″-p-coumaryl-6″-ferulylglucoside), 10: Kaempferol-7-O-glucoside, 11: Piceatannol, 12: Procyanidin B, 13: Procyanidin B6, 14: Resveratrol, 15: Picealactone C, 16: Isolariciresinol, 17: Picealactone A, 18: Dehydroabietic acid, 19: 12-Hydroxydehydroabietic acid, 20: Abieta-8,11,13-trien-7-one, 21: 7-Oxodehydroabietic acid, 22: Abietic acid, 23: Sandaracopimaric acid, 24: Typhasterol, 25: (+)-Picealactone B, 26: (+)-Lariciresinol, 27: Gibberellin A1, 28: Gibberellin A15, 29: Gibberellin A51, 30: Gibberellin A29, 31: Gibberellin A7, 32: Neocembrene. (PDF 131 kb)

Additional file 5: **Figure S5.** Total ion chromatograms obtained from (A) needles, (B) bark and (C) cones by ESI-QTOF positive electrospray ionization. (PDF 1006 kb)

Additional file 6: **Figure S6.** Taxifolin protects PC12 cells from high glucose-mediated death. Exposure to glucose toxicity produced a significant loss in cell viability compared to normal glucose conditions as assessed by mitochondrial dehydrogenase activity measured by cleavage of the formazan dye WST [left panel, ** denotes a significant difference (*p* < 0.01) relative to normal glucose control, Student’s t-test, *n* = 16]. Three concentrations of taxifolin (0.8, 1.6 and 3.2 μM) were administered to high glucose-treated cells. Cytoprotective activity was assessed as described in methods section. *denote significant differences (*p* < 0.05) between high glucose and taxifolin sample (ANOVA, post-hoc Dunnett’s t-test, *n* = 10–15). Data are reported as the mean ± SEM. (PDF 618 kb)

## Data Availability

The data and materials used in this study are available from the corresponding author on request.

## Abbreviations

ATCC: American Type Culture Collection
DPPH: 1,1-diphenyl-2-picryl-hydrazyle radical
SIV: Syndromic Importance Value
UPLC-QTOF MS: Ultra-Performance Liquid Chromatography-quadrupole time-of-flight mass spectrometry
