# Supplementary figures and images for: Growth environment and organ specific variation in in-vitro cytoprotective activities of Picea mariana in PC12 cells exposed to glucose toxicity: a plant used for treatment of diabetes symptoms by the Cree of Eeyou Istchee (Quebec, Canada)
Source: BMC Complement Altern Med. 2019 Jun 18;19:137. doi: 10.1186/s12906-019-2550-4 (PMC6582571; doi:10.1186/s12906-019-2550-4)

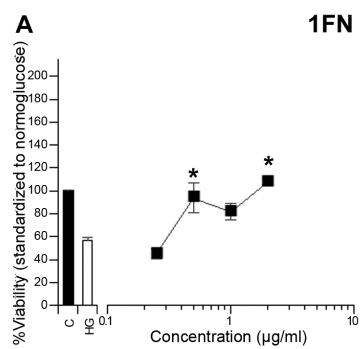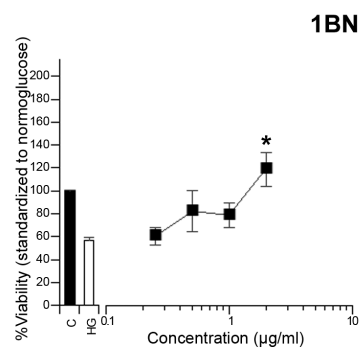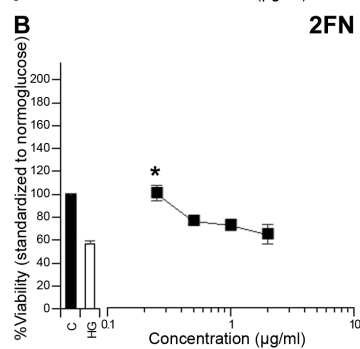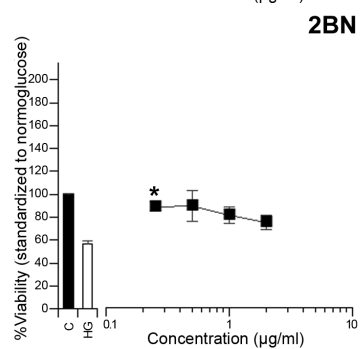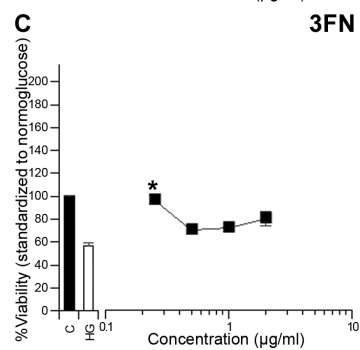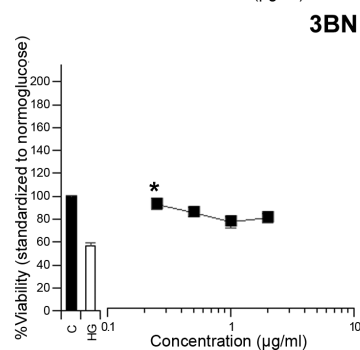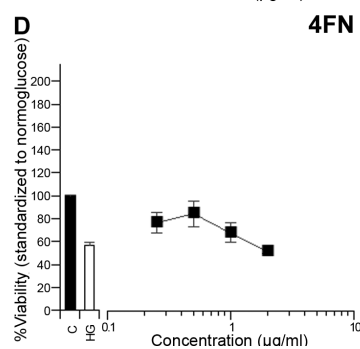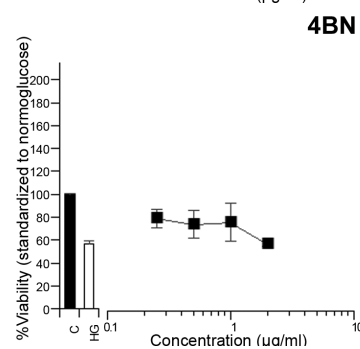

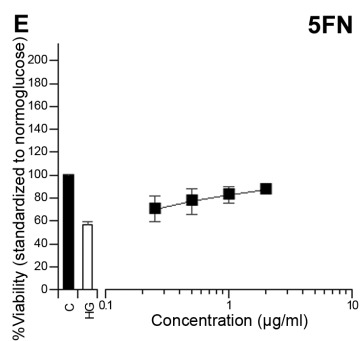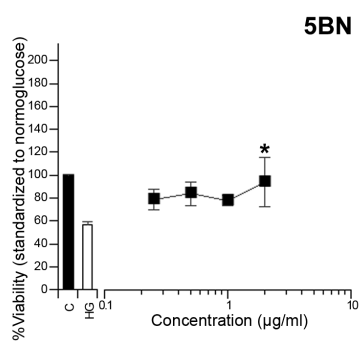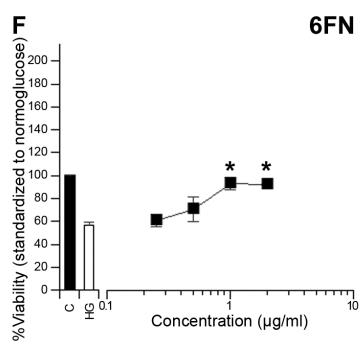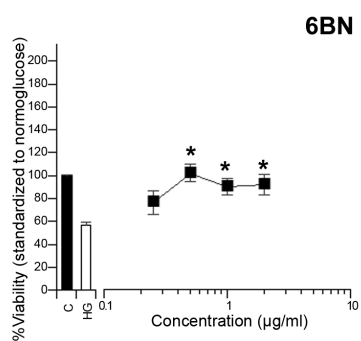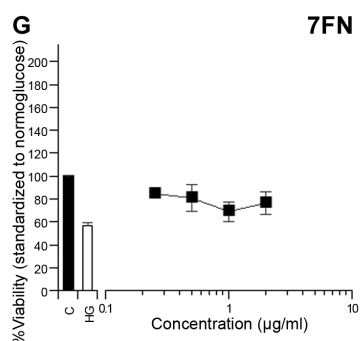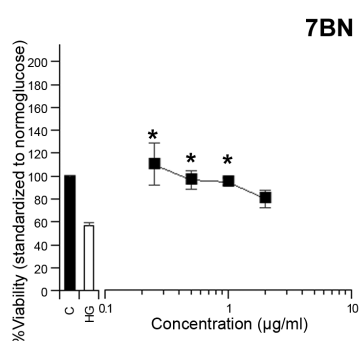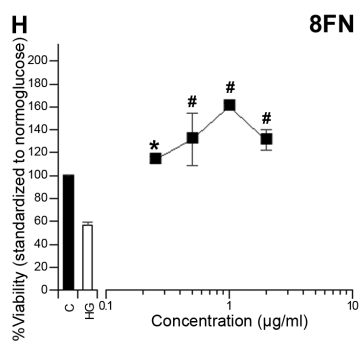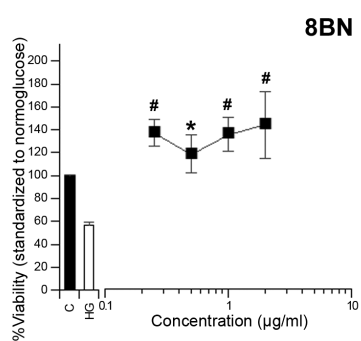

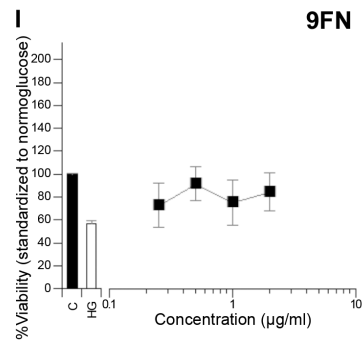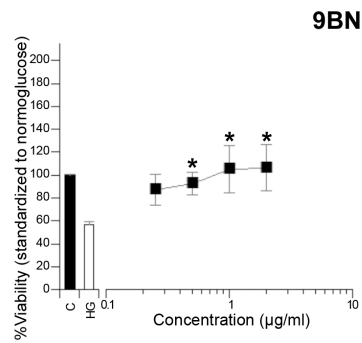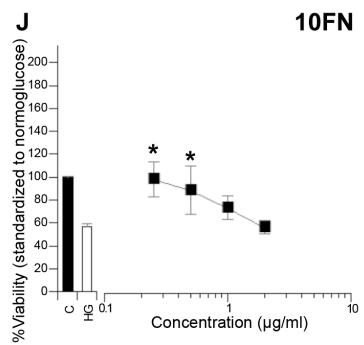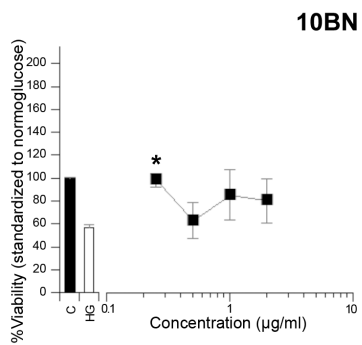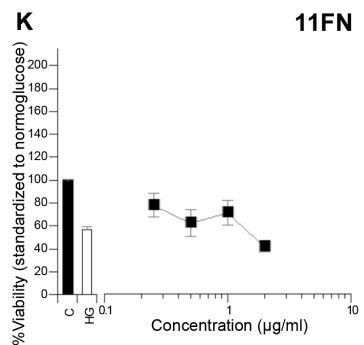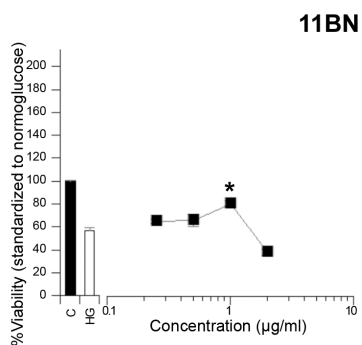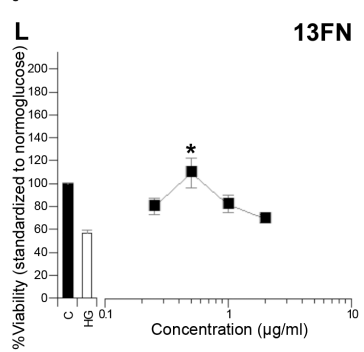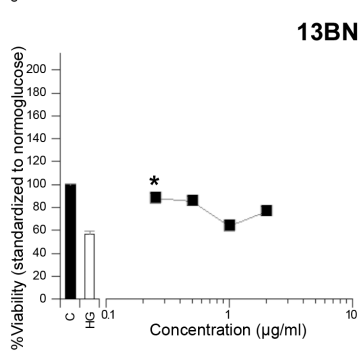

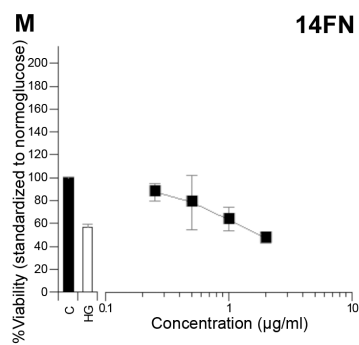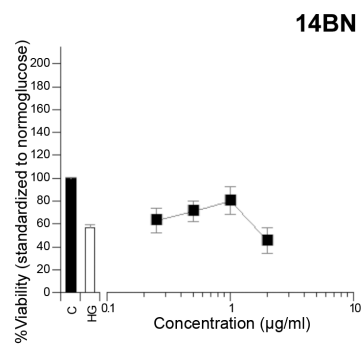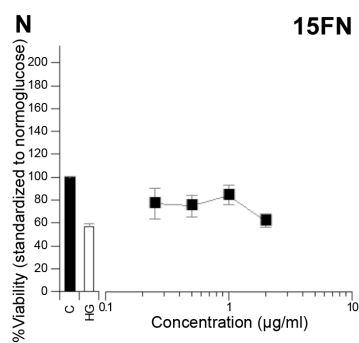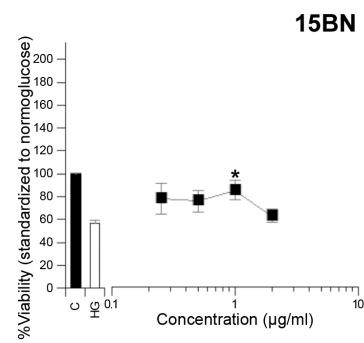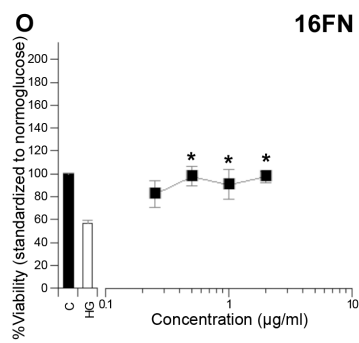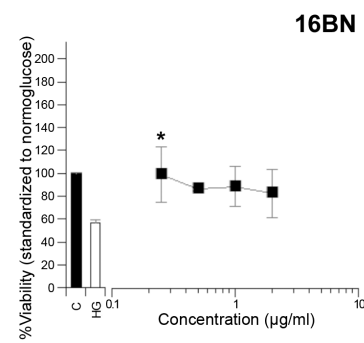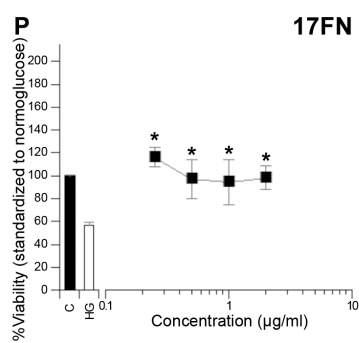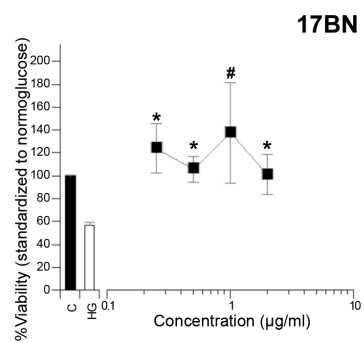

**Q 18FN**

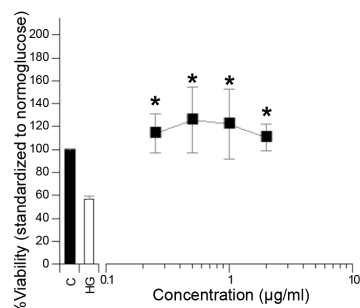

**18BN**

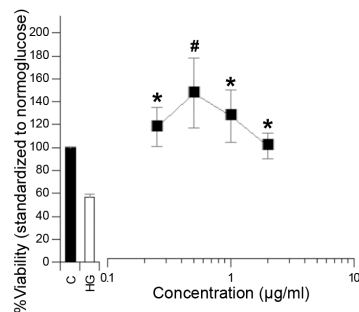

**R 19FN**

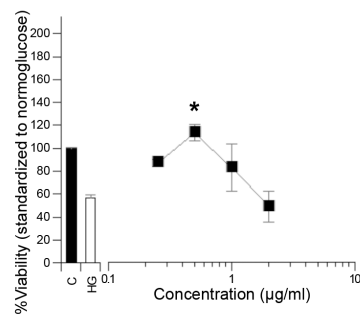

**19BN**

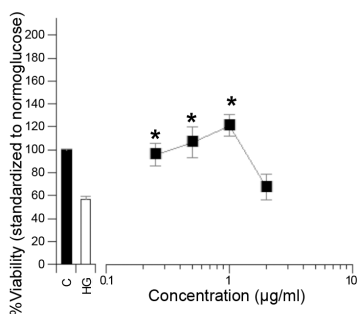

**S 20FN**

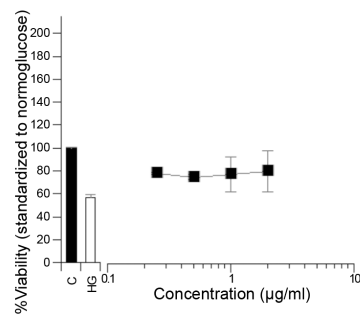

**20BN**

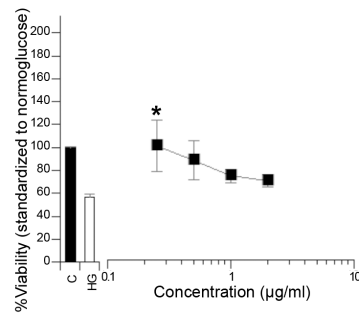

**T 21FN**

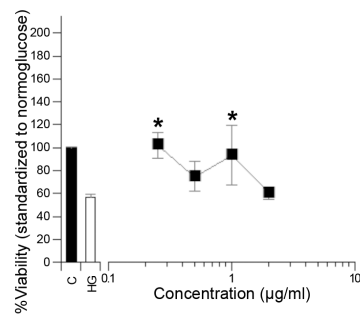

**21BN**

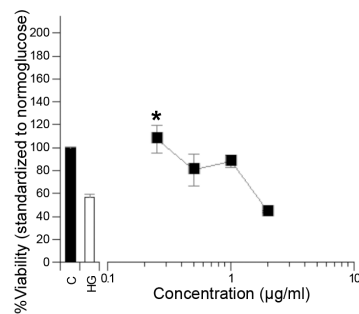

Supplement: Supplementary file 1 — Figure S1. (A-T). Comparison of cytoprotective, mitogenic and cytotoxic activities of P. mariana pooled needle extracts from forest and bog populations in a PC12-AC model of high glucose stress. Populations are listed in ascending order from coastal west [1] to inland east [21] with forest (F) and bog (B) populations next to one another for comparison. Organ type is specified as the last number in the code; needle (N). Therefore, each graph has a code identifying population number, habitat type and organ type above it in that order. Bioactivity was assessed using the formazan dye WST which measures mitochondrial dehydrogenase activity. Treatment wells containing extract were standardized to the normoglucose control (C) and compared to this and the high glucose control (HG) for the determination of protective, mitogenic or toxic effects. A students t-test was used in order to determine the significant difference between the normoglucose control (100%) and the high glucose control (56.799%, n = 54 wells/condition, bar graph, p ≤ 0.05). Anova analysis was employed in order to compare the % viability for each concentration 0.25, 0.50, 1.00 and 2.00 μg/mL to the normoglucose and high glucose controls. Differences were deemed significant (*cytoprotection, #mitogenic) when p ≤ 0.05 (n = 3 treatment wells/concentration). (PDF 973 kb) [file 12906_2019_2550_MOESM1_ESM.pdf]

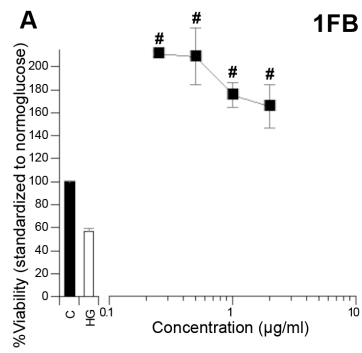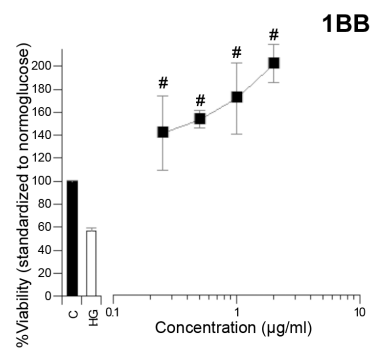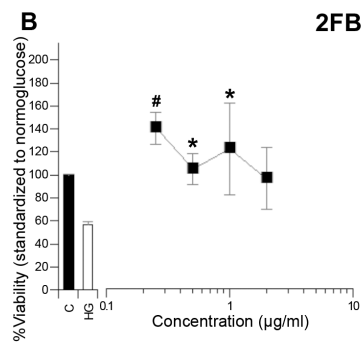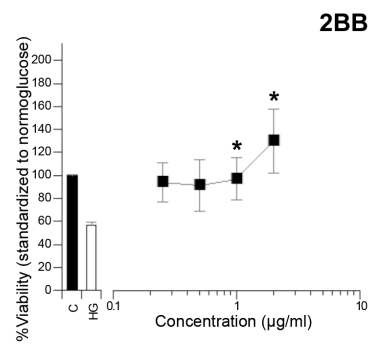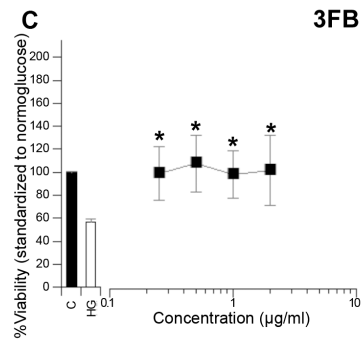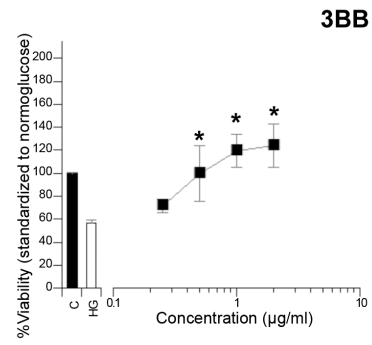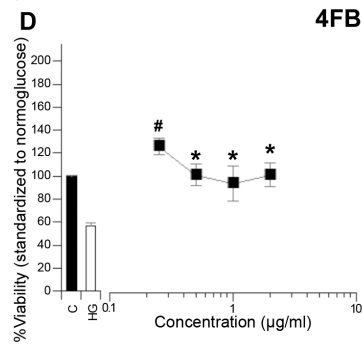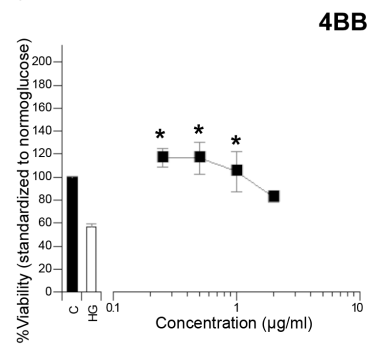

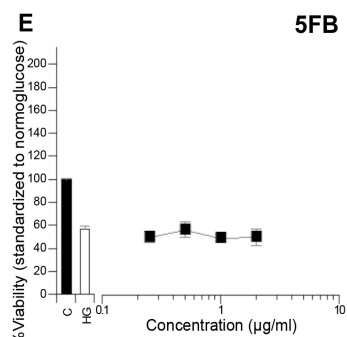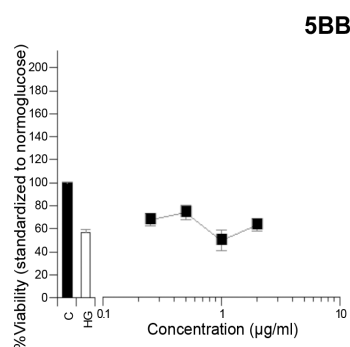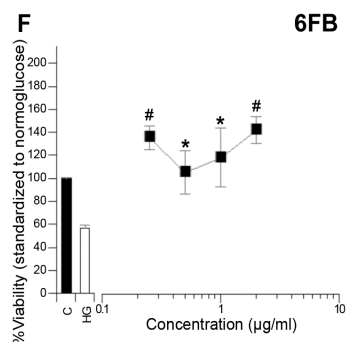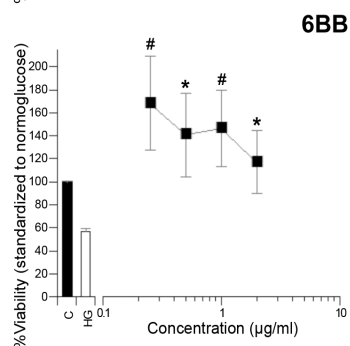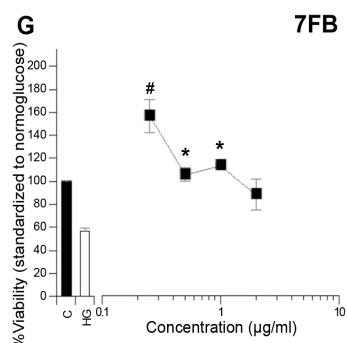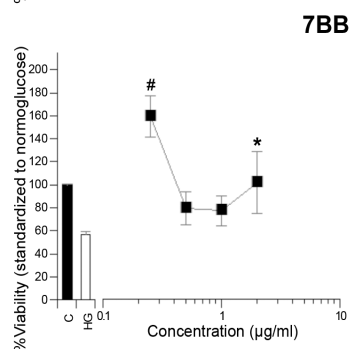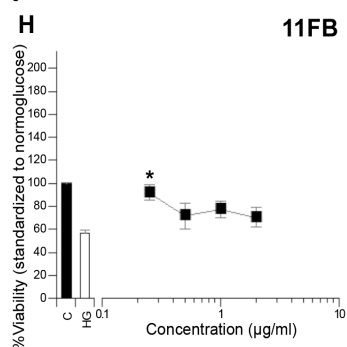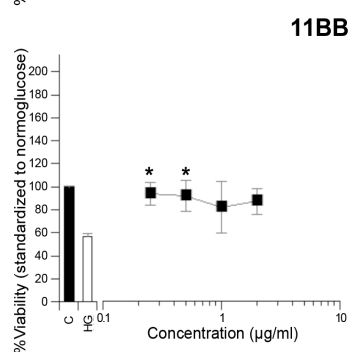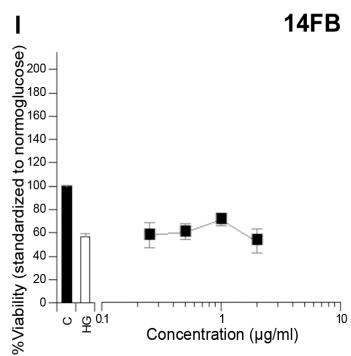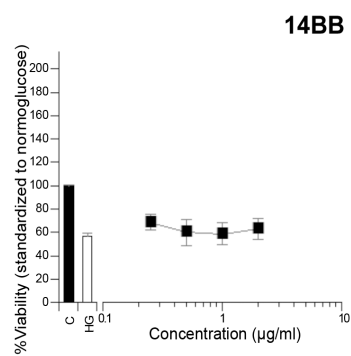

Supplement: Supplementary file 2 — Figure S2. (A-I). Comparison of cytoprotective, mitogenic and cytotoxic activities of P. mariana pooled bark extracts from forest and bog populations in a PC12-AC model of high glucose stress. Populations are listed in ascending order from coastal west [1] to inland east [14] with forest (F) and bog (B) populations next to one another for comparison. Organ type is specified as the last number in the code; bark (B). Therefore each graph has a code identifying population number, habitat type and organ type above it in that order. Bioactivity was assessed using the formazan dye WST which measures mitochondrial dehydrogenase activity. Treatment wells containing extract were standardized to the normoglucose control (C) and compared to this and the high glucose control (HG) for the determination of protective, mitogenic or toxic effects. A students t-test was used in order to determine the significant difference between the normoglucose control (100%) and the high glucose control (56.799%, n = 54 wells/condition, bar graph, p ≤ 0.05). Anova analysis was employed in order to compare the % viability for each concentration 0.25, 0.50, 1.00 and 2.00 μg/mL to the normoglucose and high glucose controls. Differences were deemed significant (*cytoprotection, #mitogenic) when p ≤ 0.05 (n = 3 treatment wells/concentration). (PDF 476 kb) [file 12906_2019_2550_MOESM2_ESM.pdf]

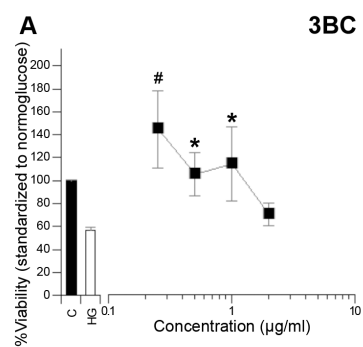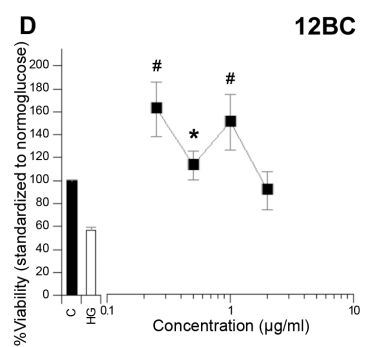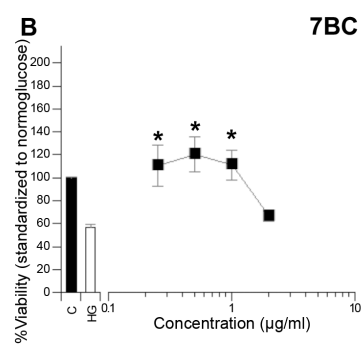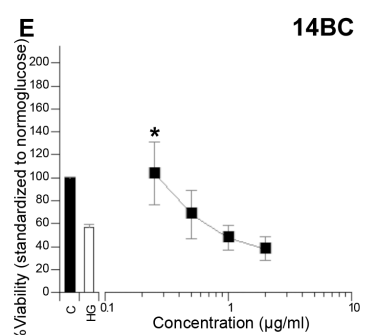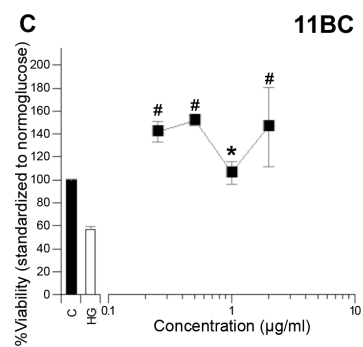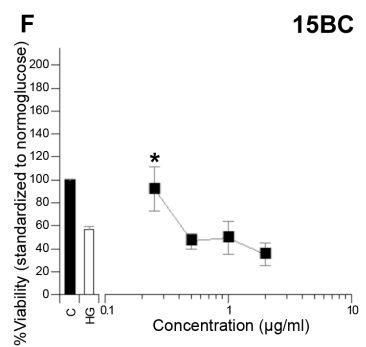

Supplement: Supplementary file 3 — Figure S3. (A-F). Comparison of cytoprotective, mitogenic and cytotoxic activities of P. mariana pooled cone extracts from bog populations in a PC12-AC model of high glucose stress. Populations are listed in ascending order from coastal west [3] to inland east [15] Habitat is the second letter in the code (bog = B) while Organ type is specified as the last number in the code (cone = C). Therefore each graph has a code identifying population number, habitat type and organ type above it in that order. Bioactivity was assessed using the formazan dye WST which measures mitochondrial dehydrogenase activity. Treatment wells containing extract were standardized to the normoglucose control (C) and compared to this and the high glucose control (HG) for the determination of protective, mitogenic or toxic effects. A students t-test was used in order to determine the significant difference between the normoglucose control (100%) and the high glucose control (56.799%, n = 54 wells/condition, bar graph, p ≤ 0.05). Anova analysis was employed in order to compare the % viability for each concentration 0.25, 0.50, 1.00 and 2.00 μg/mL to the normoglucose and high glucose controls. Differences were deemed significant (*cytoprotection, #mitogenic) when p ≤ 0.05 (n = 3 treatment wells/concentration). (PDF 171 kb) [file 12906_2019_2550_MOESM3_ESM.pdf]

A:

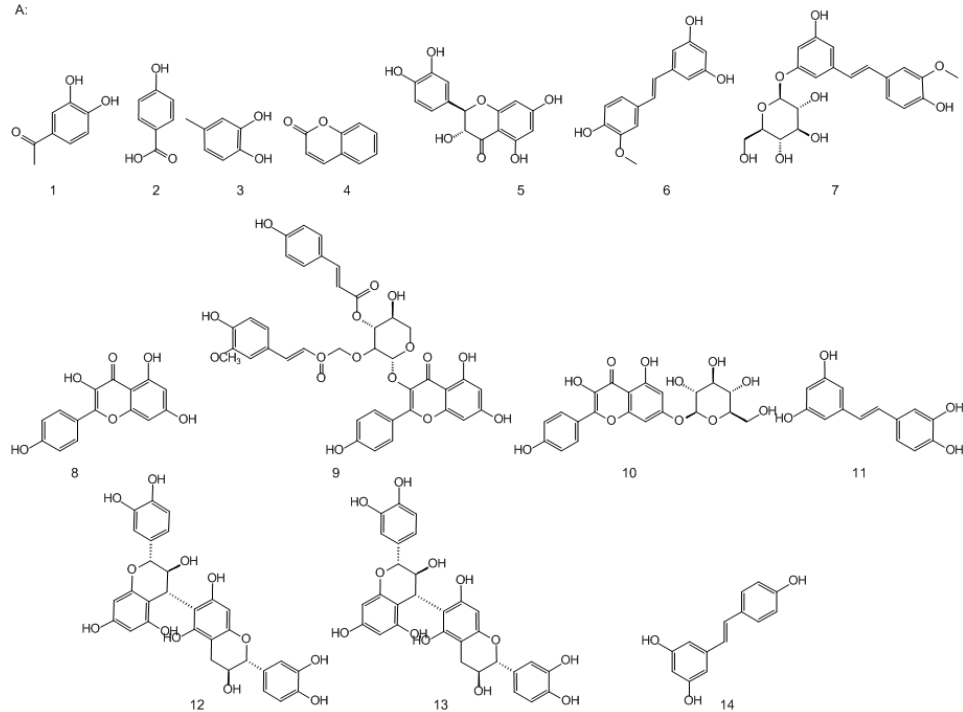

B:

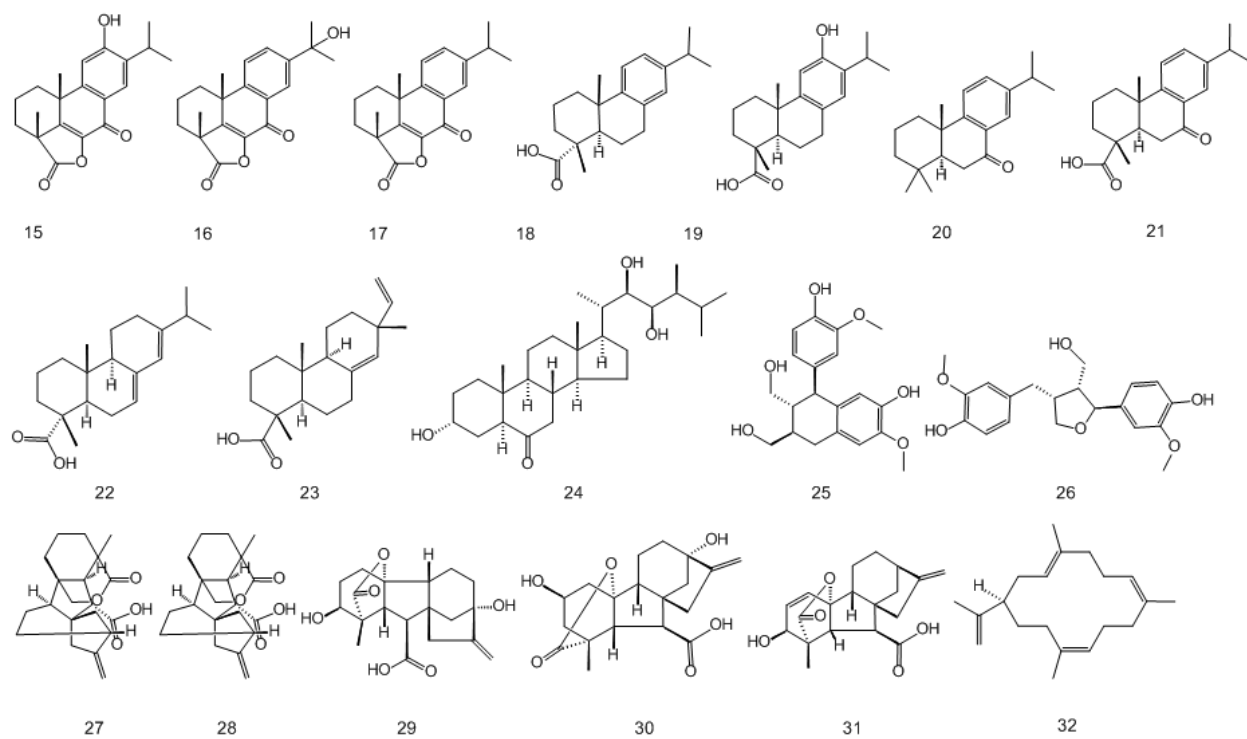

Supplement: Supplementary file 4 — Figure S4. (A-B). Chemical structures of compounds in P. mariana needles, bark and cone extracts identified by ESI-QTOF positive electrospray ionization. (A) Phenolics, (B) Terpenes. 1: 3′,4′-Dihydroxyacetophenone, 2: 4-Hydroxybenzoic acid, 3: 4-Methylcatechol, 4: Coumarin, 5: Dihydroquercetin, 6: Isorhapontigenin, 7: Isorhapontigenin 3-O-beta-D-glucopyranoside, 8: Kaempferol, 9: Kaempferol 3-(3″-p-coumaryl-6″-ferulylglucoside), 10: Kaempferol-7-O-glucoside, 11: Piceatannol, 12: Procyanidin B, 13: Procyanidin B6, 14: Resveratrol, 15: Picealactone C, 16: Isolariciresinol, 17: Picealactone A, 18: Dehydroabietic acid, 19: 12-Hydroxydehydroabietic acid, 20: Abieta-8,11,13-trien-7-one, 21: 7-Oxodehydroabietic acid, 22: Abietic acid, 23: Sandaracopimaric acid, 24: Typhasterol, 25: (+)-Picealactone B, 26: (+)-Lariciresinol, 27: Gibberellin A1, 28: Gibberellin A15, 29: Gibberellin A51, 30: Gibberellin A29, 31: Gibberellin A7, 32: Neocembrene. (PDF 131 kb) [file 12906_2019_2550_MOESM4_ESM.pdf]

**A**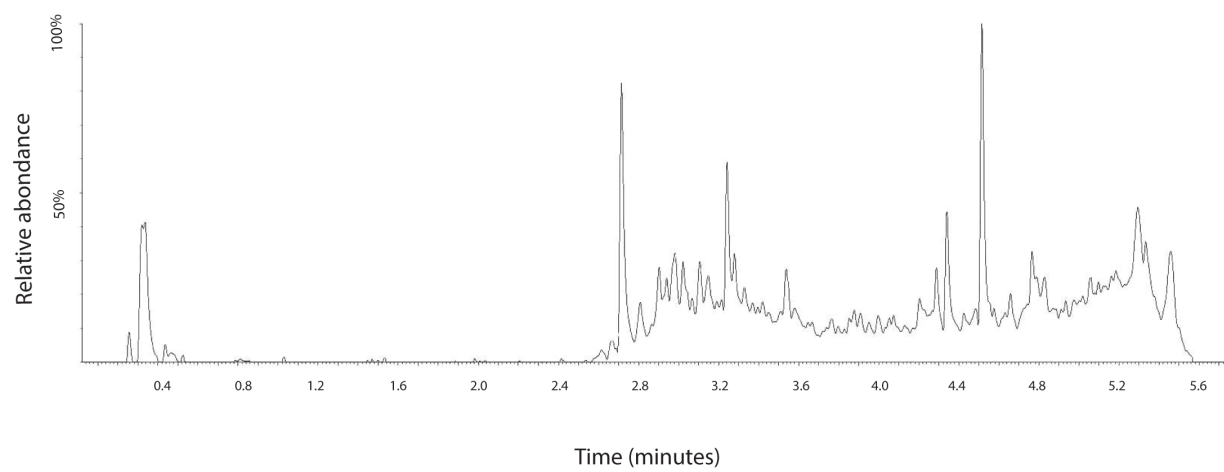**B**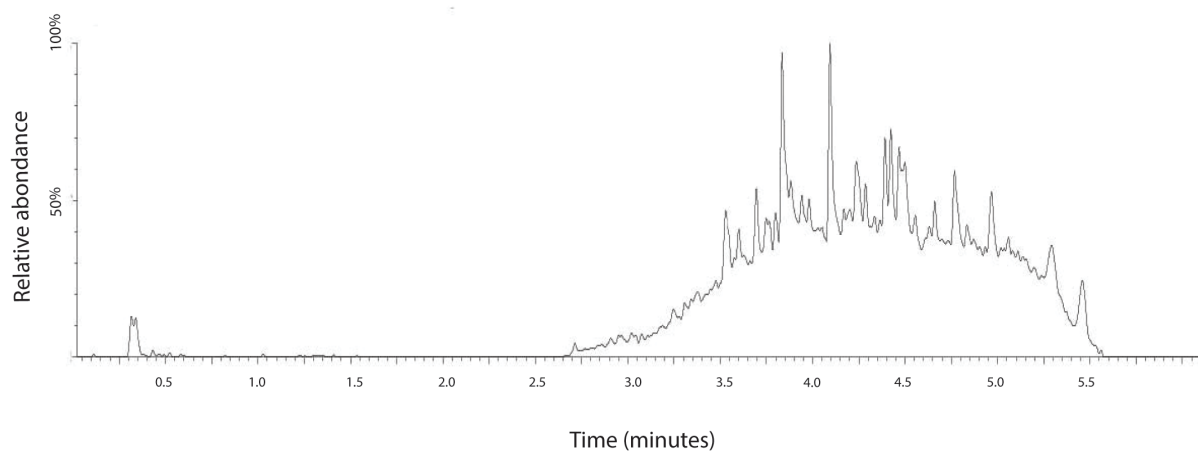**C**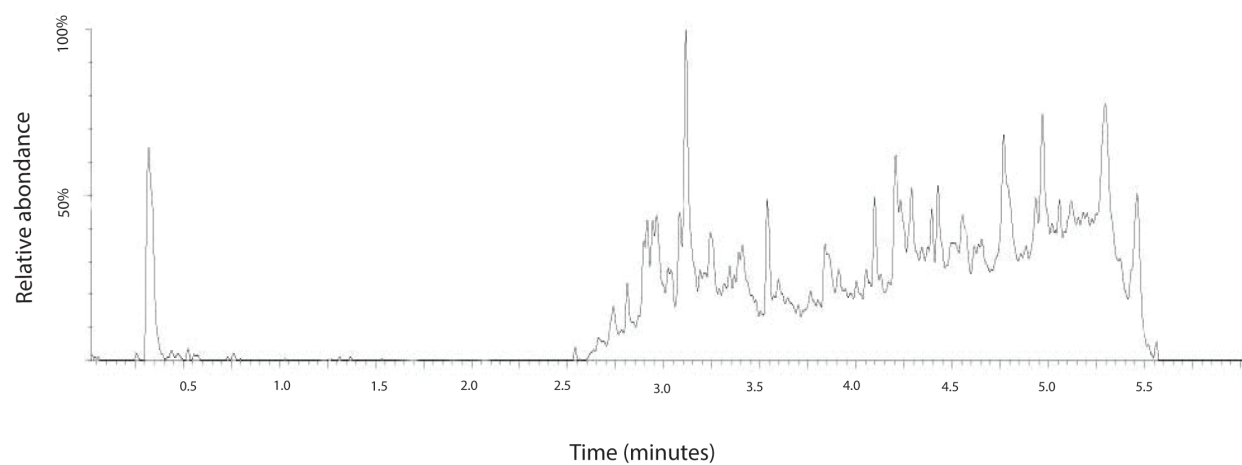

Supplement: Supplementary file 5 — Figure S5. Total ion chromatograms obtained from (A) needles, (B) bark and (C) cones by ESI-QTOF positive electrospray ionization. (PDF 1006 kb) [file 12906_2019_2550_MOESM5_ESM.pdf]

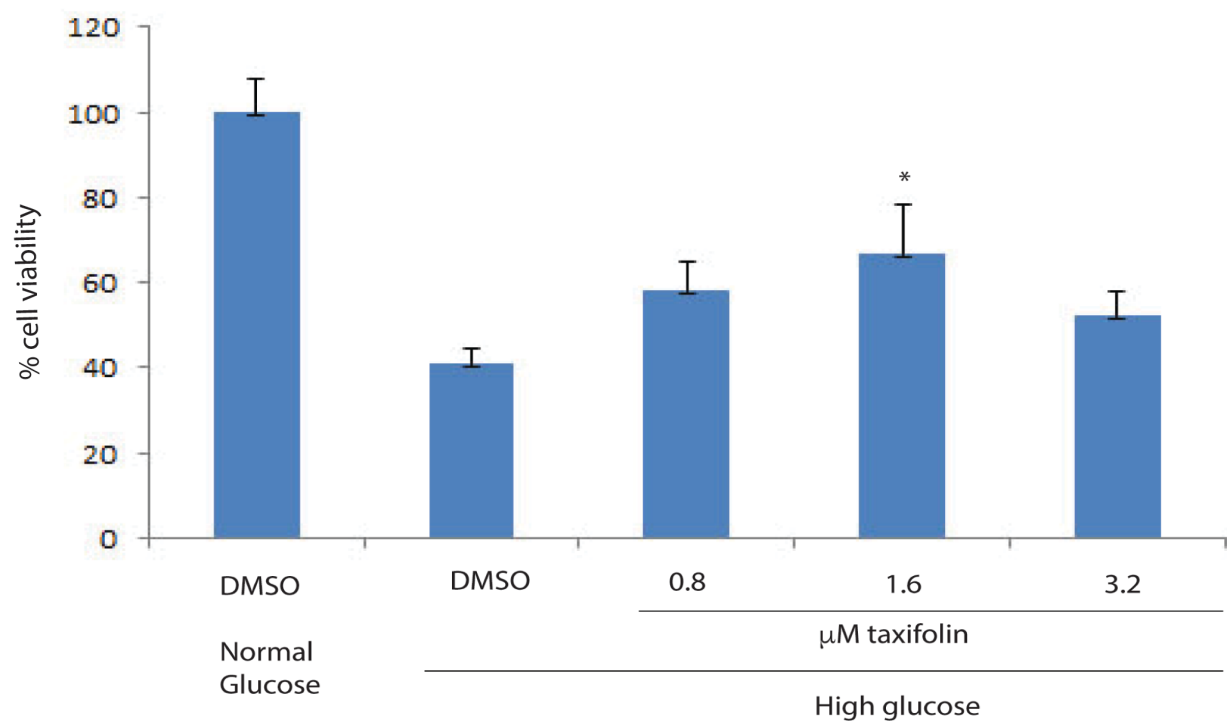

Supplement: Supplementary file 6 — Figure S6. Taxifolin protects PC12 cells from high glucose-mediated death. Exposure to glucose toxicity produced a significant loss in cell viability compared to normal glucose conditions as assessed by mitochondrial dehydrogenase activity measured by cleavage of the formazan dye WST [left panel, ** denotes a significant difference (p < 0.01) relative to normal glucose control, Student’s t-test, n = 16]. Three concentrations of taxifolin (0.8, 1.6 and 3.2 μM) were administered to high glucose-treated cells. Cytoprotective activity was assessed as described in methods section. *denote significant differences (p < 0.05) between high glucose and taxifolin sample (ANOVA, post-hoc Dunnett’s t-test, n = 10–15). Data are reported as the mean ± SEM. (PDF 618 kb) [file 12906_2019_2550_MOESM6_ESM.pdf]
